# Targeting macrophagic PIM-1 alleviates osteoarthritis by inhibiting NLRP3 inflammasome activation via suppressing mitochondrial ROS/Cl^−^ efflux signaling pathway

**DOI:** 10.1186/s12967-023-04313-1

**Published:** 2023-07-08

**Authors:** Zhen Zhang, Shujun Xie, Jin Qian, Fengqiang Gao, Wenjian Jin, Lingqiao Wang, Lili Yan, Hao Chen, Wangxiang Yao, Maoqiang Li, Xuepeng Wang, Liulong Zhu

**Affiliations:** 1 Department of Orthopedics Surgery, Affiliated Hangzhou First People’s Hospital, Zhejiang University School of Medicine, Hangzhou, 31000 Zhejiang China; 2 Department of Translational Medicine Research Center, Key Laboratory of Clinical Cancer Pharmacology and Toxicology Research of Zhejiang Province, Affiliated Hangzhou First People’s Hospital, Zhejiang University School of Medicine, Cancer Center, Zhejiang University, 310006 Hangzhou, China; 3 Department of Hepatobiliary and Pancreatic Surgery, Affiliated Hangzhou First People’s Hospital, Zhejiang University School of Medicine, Hangzhou, China; 4 Department of Hepatobiliary Surgery, The Third Affiliated Hospital of Soochow University, Changzhou, 213000 China; 5 Department of Pediatrics, Affiliated Hangzhou First People’s Hospital, Zhejiang University School of Medicine, Hangzhou, 31000 Zhejiang China

**Keywords:** PIM-1, SMI-4a, NLRP3 inflammasome, Mitochondrial ROS, Chloride efflux, Macrophages, Chondrocytes, Osteoarthritis

## Abstract

**Background:**

Osteoarthritis (OA), in which macrophage-driven synovitis is considered closely related to cartilage destruction and could occur at any stage, is an inflammatory arthritis. However, there are no effective targets to cure the progression of OA. The NOD-, LRR-,and pyrin domain-containing protein 3 (NLRP3) inflammasome in synovial macrophages participates in the pathological inflammatory process and treatment strategies targeting it are considered to be an effective approach for OA. PIM-1 kinase, as a downstream effector of many cytokine signaling pathways, plays a pro-inflammatory role in inflammatory disease.

**Methods:**

In this study, we evaluated the expression of the PIM-1 and the infiltration of synovial macrophages in the human OA synovium. The effects and mechanism of PIM-1 were investigated in mice and human macrophages stimulated by lipopolysaccharide (LPS) and different agonists such as nigericin, ATP, Monosodium urate (MSU), and Aluminum salt (Alum). The protective effects on chondrocytes were assessed by a modified co-culture system induced by macrophage condition medium (CM). The therapeutic effect in vivo was confirmed by the medial meniscus (DMM)-induced OA in mice.

**Results:**

The expression of PIM-1 was increased in the human OA synovium which was accompanied by the infiltration of synovial macrophages. In vitro experiments, suppression of PIM-1 by SMI-4a, a specific inhibitor, rapidly inhibited the NLRP3 inflammasome activation in mice and human macrophages and gasdermin-D (GSDME)-mediated pyroptosis. Furthermore, PIM-1 inhibition specifically blocked the apoptosis-associated speck-like protein containing a CARD (ASC) oligomerization in the assembly stage. Mechanistically, PIM-1 inhibition alleviated the mitochondrial reactive oxygen species (ROS)/chloride intracellular channel proteins (CLICs)-dependent Cl^−^ efflux signaling pathway, which eventually resulted in the blockade of the ASC oligomerization and NLRP3 inflammasome activation. Furthermore, PIM-1 suppression showed chondroprotective effects in the modified co-culture system. Finally, SMI-4a significantly suppressed the expression of PIM-1 in the synovium and reduced the synovitis scores and the Osteoarthritis Research Society International (OARSI) score in the DMM-induced OA model.

**Conclusions:**

Therefore, PIM-1 represented a new class of promising targets as a treatment of OA to target these mechanisms in macrophages and widened the road to therapeutic strategies for OA.

**Supplementary Information:**

The online version contains supplementary material available at 10.1186/s12967-023-04313-1.

## Introduction

Synovitis is considered closely related to cartilage destruction and could occur at any stage of osteoarthritis (OA) [1, 2]. Inflammatory cytokines produced by synovial macrophages promote cartilage damage [3, 4]. Targeting synovial macrophages for the therapeutic of OA is receiving increased attention for the past few years [3, 4]. The NOD-, LRR-,and pyrin domain-containing protein 3 (NLRP3) is an intracellular sensor that detects pathogen-associated molecular patterns (PAMPs) and damage-associated molecular patterns (DAMPs), resulting in the formation and activation of the NLRP3 inflammasome [5]. NLRP3 inflammasome is a multi-protein complex that is primarily found in macrophages and promotes a myriad of inflammatory cytokines secretion, such as IL-1β [6]. It was found that the activation of NLRP3 inflammasome was enhanced in the synovium of OA [7, 8]. Therefore, the NLRP3 inflammasome-targeted therapy is supposed promising treatment method for OA [9, 10].

NLRP3 inflammasome is activated by two steps: the priming stage and the assembly stage [11]. The priming stage is initiated by lipopolysaccharide (LPS), which upregulates NF-κB-induced NLRP3 and pro-IL-1β protein expression [12]. At this stage, NLRP3 is stabilized in a signal-competent and auto-suppressed inactive state [13]. In the assembly stage, NLRP3 inflammasome activation is initiated when NLRP3 is exposed to PAMPs and DAMPs. Subsequently, NLRP3 protein oligomerizes and recruits apoptosis-associated speck-like protein containing a CARD (ASC) protein to continue nucleation to form ASC oligomers [14]. The inflammatory cysteine protease caspase-1 is activated by the ASC complex and enables the cleavage of pro-IL-1β and gasdermin-D (GSDMD) into IL-1β and N-terminal fragment of GSDMD (N-GSDMD) [15]. Finally, N-GSDMD forms transmembrane pores to secrete mature IL-1β and to drive macrophage pyroptosis [16, 17]. The joint cavity presents various DAMPs such as cartilage fragments, extracellular matrix (ECM) proteins, ATP, and Monosodium urate (MSU), which could mediate NLRP3 inflammasome assembly and activation [18, 19].

PIM-1 kinase, as a downstream effector of many cytokine signaling pathways, is triggered by many cytokines (such as IL-3, IL-6, and TNF-a) through the regulation of the JAK/STAT signaling [20–23]. It can be inhibited by specific kinase inhibitors such as SMI-4a, and the proapoptotic Bcl-2–associated agonist of cell death (Bad) is considered its downstream target [24–26]. PIM-1 plays a pro-inflammatory role and the function of PIM-1 has been extensively studied in inflammatory diseases [27]. SMI-4a, a benzylidene-thiazolidene-2,4-dione, showed remarkable anti-inflammatory effects on animal models through the suppression of PIM-1. It has been shown that SMI-4a exhibited inhibitory functions on PIM-1/ZEB1/E-cadherin pathway to mitigate bleomycin-induced pulmonary fibrosis [28]. Besides, it exerts a protective effect against acute lung injury through the regulation of the PIM-1/ELK3/ICAM1 axis [29]. Previous studies have found that PIM-1 was highly expressed in macrophages [22]. Of note, the number of macrophages and inflammatory factor expression increased in the synovium of OA [30]. However, the role of PIM-1 in OA synovial macrophages remains ambiguous. ATP and MSU can promote the production of mitochondrial reactive oxygen species (ROS), which is one of the critical mediators of NLRP3 inflammasome assembly and activation [31–34]. Intriguingly, PIM-1 plays a role in the regulation of mitochondrial ROS [35]. Moreover, PIM-1 kinase is related to NLRP3 inflammasome activation [24, 36, 37]. However, whether PIM-1 participates in the assembly stage of NLRP3 inflammasome activation and whether the potential function is mediated by mitochondrial ROS remains to be explored. Besides, whether PIM-1 inhibition can produce the protection effect on chondrocytes remains unclear.

Here, we found that macrophage infiltration and PIM-1 expression had a significant overall rise in the human OA synovium. Moreover, SMI-4a, a specific kinase inhibitor could specifically alleviate ASC oligomerization to inhibit the NLRP3 inflammasome in the assembly stage. Moreover, inhibiting PIM-1 blocked the ASC oligomerization via the mitochondrial ROS/Cl^−^ efflux pathway. Additionally, PIM-1 suppression could possess chondroprotective effects in a modified co-culture system. Finally, SMI-4a ameliorated synovium inflammation and cartilage damage in mice destabilization of the medial meniscus (DMM)-induced OA models. To sum up, PIM-1 represented a new class of therapeutic targets for synovitis and widened the road to therapeutic strategies for OA.

## Materials and methods

### Cell culture and stimulation

Peritoneal macrophages (PMs): The 6-8-week-old male C57BL/6 mice were injected intraperitoneally with 2 ml of 4% fluid thioglycollate medium (Merck, Darmstadt, Germany). After 5 days, the mice were sacrificed, and PMs were obtained from their peritoneal cavity. Briefly, the peritoneal cavity was washed with PBS buffer (PBS + 5% FBS + 2 mM Ethylenediaminetetraacetic acid), and the peritoneal fluid was collected, filtered through a 70-µm-pore-diameter nylon mesh. After centrifuging, the pellet was first resuspended with red blood cell lysis buffer. After five minutes, the process was stopped by adding PBS buffer. The solution was centrifuged and the pellet was resuspended with DMEM medium. The PM-containing solution was plated onto DMEM medium supplemented with 10% FBS, and 1% PS.

Bone marrow-derived macrophages (BMDMs): Mouse femur and tibia were obtained from the limbs. The medullary cavity of the C57BL/6 mice was flushed with the solution (PBS + 2% FBS) using a 1 ml needle. The medullary cavity flush was collected and filtered through a 70-µm-pore diameter nylon mesh and centrifuged. The pellet was first resuspended with the red blood cell lysis buffer for 5 min and resuspended with 3 x volume of PBS buffer. After centrifuging, the pellet was resuspended with DMEM medium. The cell-containing solution was plated onto DMEM plates supplemented with 10% FBS, 1% PS, and 10 ng/mL murine macrophage colony-stimulating factor (M-CSF) (Novoprotein, Suzhou, China). The cells were differentiated with 10 ng/ml of M-CSF for 5 days to generate BMDMs. After 5 days of differentiation, the BMDM cells were transferred into 12-well plates at a density of 5 × 10^6^ cells per well and cultured overnight.

The following day, the medium was replaced with Opti-MEM (InvivoGen, San Diego, CA, USA) supplement with 1 µg/ml of LPS (Sigma, MO, USA) and cultured for 4 h to induce the production of NLRP3 and pro-IL-1β.

To activate the NLRP3 inflammasome, the cells in the induced medium were divided and stimulated separately with either 10 µM of nigericin (InvivoGen, CA, USA) for 45 min, or 2.5 mM of ATP (Sigma, MO, USA) for 45 min, or 300 µg/ml of MSU (InvivoGen, CA, USA) for 3 h, or 320 µg/ml of Aluminum salts (Alum) (Thermo Fisher Scientific, MA, USA) for 3 h.

To activate the absent in melanoma 2 (AIM2) inflammasome, the cells were treated with 1 µg/ml of poly (dA:dT) employing Lipofectamine 2000 (Invitrogen, CA, USA) for 45 min. To activate the NLR family, CARD domain-containing protein 4 (NLRC4) inflammasome, the cells were infected with Salmonella typhimurium (Salmonella) for 1 h. The Salmonella culture was centrifuged, and then was added into the medium of BMDMs (1: 100). Finally, the cells were treated with gentamycin for 1 h.

The PIM-1 kinase inhibitors SMI-4a and AZD1208 were purchased from Selleck Chemicals (TX, USA) and solubilized in dimethyl sulfoxide (DMSO) at 30 µM.

### ELISA

The macrophage culture was collected and centrifuged (3000 rpm, 4 °C, 5 min). The supernatants were diluted five-fold for IL-1β detection and ten-fold for TNF-a detection. IL-1β and TNF-a were analyzed with an ELISA kit (InvivoGen, CA, USA) according to the manufacturer’s guideline.

### LDH release assay

The BMDMs were stimulated with LPS (1 µg/mL) for 4 h, and then co-incubated with SMI-4a at different doses (3.3 μm, 10 μm, 30 μm), followed by 45 min of nigericin stimulation. The culture medium was collected and centrifuged at 1000 × g for 5 min to remove cellular debris. The supernatant was used to measure the activity of lactate dehydrogenase (LDH) (Beyotime, Shanghai, China).

### Western blotting

The total proteins from different macrophages and chondrocytes were extracted with the RIPA lysis buffer (MCE, NJ, USA) containing a mixture of phosphatase and protease inhibitors. Then, the proteins were separated with 8–12% SDS-PAGE (Febio science, Hangzhou, China) and transferred to the polyvinylidene fluoride membranes (Merk Millipore, Darmstadt, Germany). The membranes were blocked with 5%BSA and then incubated with the primary antibodies overnight. The membranes were washed next day with PBST and then incubated with the HRP-conjugated secondary antibodies (Beyotime, Shanghai, China) for 90 min. These results on the membranes were captured and documented by Molecular Imager (Bio-Rad, CA, USA).

The primary antibodies used include those against IL-1β (R&D Systems, MN, USA), NLRP3 (Adipogen, CA, USA), ASC (Adipogen), Caspase-1 (Adipogen), P-jak2 (Abcam, MA, USA), P-stat3 (Abcam), P-bad (Cell Signaling Technology, MA, USA), Cleaved Caspase-3 (Cell Signaling Technology), Jak2 (ABclonal, Wuhan, China), Stat3 (ABclonal), Bad (ABclonal), Clic1 (ABclonal), Clic4 (ABclonal), ADAMTS5 (ABclonal), MMP13 (ABclonal), Bax (HUABIO, Hangzhou, China), Bcl-2 (HUABIO), Clic5 (HUABIO), PIM-1 (HUABIO), Collagen II (ProteinTech, Wuhan, China), Aggrecan (ProteinTech), β-actin (ProteinTech), GADPH (ProteinTech).

### The formation of ASC oligomers

The BMDMs were treated with 1 µg/ml LPS for 4 h and then stimulated with 10 µM nigericin for 45 min. Next, the cells were washed with PBS and submerged in 500 µl buffer (50 mM Tris–HCl, 0.5% Triton X-100, 1 mM DL-dithiothreitol, and 1 mM Phenylmethylsulfonyl fluoride). Then, the cells were suspended in 1.5ml Eppendorf tubes by pipetting 20 times with a 21-gauge needle. The cell suspensions were centrifuged (3300 g, 10 min, 4 °C). The precipitates were washed with PBS and incubated with 4 mM disuccinimidyl suberate (DSS) (Sigma) by rotation for 30 min. Then, the mixed liquid was centrifuged (3300 g, 10 min, 4 °C), and the pellet was dissolved in 40 µL of 2×loading buffer (Beyotime, Shanghai, China).

### Potassium and chloride replacement

The BMDMs were cultured in Opti-MEM with LPS (1 ug/ml) for 4 h. Then they were divided into four groups and treated separately with different isotonic salt solutions containing 1 µg/ml LPS: control solution (145 mM NaCl, 5 mM KCl), K^+^ free solution (150 mM NaCl), Cl^−^ free solution (145 mM NaGluconate, 5 mM KGluconate), or K^+^ and Cl^−^ free solution (150 mM NaGluconate) [38]. Finally, SMI-4a was added to the solutions just after replacement.

### Evaluation of the intracellular Cl^−^ level

The BMDMs were washed with PBS buffer three times. They were then treated with 10 mM of N-(Ethoxycarbonylmethyl)-6-methoxyquinolinium bromide (MQAE) (Beyotime, Shanghai, China) for 60 min and washed with PBS buffer three times. The fluorescence intensity was measured at 488 nm emission wavelength and 520 nm excitation wavelength fluorescence microscopy (Olympus, Tokyo, Japan).

### Isolation of plasma membrane fraction

The BMDMs were treated in 10-cm dishes with 1 µg/ml LPS for 4 h. The medium of one group was mixed with 10 µM nigericin containing 1 ug/ml LPS and the cells were further treated for 15 min. The medium of another group was replaced with K^+^ and Cl^−^ free solution (150 mM NaGluconate and 1 µg/ml LPS) and the cells were further treated for 30 min. The culture medium was discarded, and the cells were washed with PBS buffer three times. The plasma membranes were extracted from the cells with the Plasma Membrane Protein Extraction Kit (Abcam, MA, USA) in line with the manufacturer’s guidelines [34].

### Cartilage extraction and chondrocyte culture

The 5-day-old mice (C57BL/6) were sacrificed and disinfected. Under aseptic conditions, the femur head was exposed, and the articular cartilage was isolated. The cartilage was cut up with a scalpel into small size and incubated with 0.2% collagenase II (2 mg/ml) for 4 h. Next, the digested cartilage was resuspended in DMEM/F12 and the cell mixture was filtered through a 70 μm cell strainer and then centrifuged to obtain primary chondrocytes. These cells were seeded in DMEM/F12 and the medium was refreshed every 2 days. The second or third passage cells were utilized for the following experiments.

### Collection of macrophages conditioned medium (CM)

The BMDMs were plated in 6-well plates, treated with 1 µg/ml LPS for 4 h, and then incubated with either control solution (145 mM NaCl,5 mM KCl) or K^+^ and Cl^−^ free solution (150 mM NaGluconate) for 45 min [38]. The medium was collected and centrifuged at 3000r/min for 10 min and diluted 40 times with DMEM/F12 containing 10% fetal bovine serum for different experiments. The resulting conditioned medium was added to the chondrocytes obtained in 2.9 above for investigating the interaction of macrophages and chondrocytes.

### Chondrocyte proliferation and apoptosis analysis

The chondrocytes were incubated with the CM in 12-well plates for 48 hours for the proliferation analysis and apoptosis analysis in the co-culture system. The proliferation level was determined with 5-ethynyl-2’ -deoxyuridine (EdU) staining (Beyotime, Shanghai, China). The apoptosis level of the chondrocytes was determined with Terminal Deoxynucleotidyl Transferase-Mediated dUTP Nick-End Labeling (TUNEL) staining (Servicebio, Wuhan, China). These results were captured and documented by the fluorescence microscope (Olympus, Tokyo, Japan).

### Mice knee OA model induction

The 8-week-old C57BL/6 male mice were purchased from the Shanghai SLAC Laboratory Animal Company (Shanghai, China). The mouse OA model was established by surgical DMM after the mouse was adapted to the new environment for 2 weeks. During DMM surgery, the joint capsule was incised, and the medial meniscotibial ligament was sheared. The joint capsule of the control group was only surgically opened and then sutured the incision. Two weeks later, all mice were divided into 3 groups: Control, DMM, and DMM + SMI-4a groups at random allocation. The mice in the DMM + SMI-4a group received SMI-4a(30umol/L) through intra-articular injection of 10 ul once every two days, other groups were injected with 10 ul PBS [39]. All mice were sacrificed, and the knee joints, liver, spleen, and kidney were acquired for histological evaluation.

### Histopathological analysis

The knee joints were obtained from the OA and processed with safranin-O and fast green (S&F) and hematoxylin & eosin (H&E). For the OA model, the severity of synovitis was assessed by the synovitis scores and the severity of cartilage damage was calculated by Osteoarthritis Research Society International (OARSI) scoring system [40]. Immunohistochemical staining was accomplished with the knee joint cartilages. The primary antibodies used in the experiments were those against matrix metallopeptidase 13 (MMP13) (ABclonal), A disintegrin and metalloproteinase with thrombospondin motifs 5 (ADAMTS5) (ABclonal), Collagen II (ProteinTech), Aggrecan (ProteinTech), Cleaved Caspase-3 (Cell Signaling Technology), PIM-1(HUABIO). The images of immunohistochemical staining were captured using an Olympus VS200 system (Tokyo, Japan).

### Statistical analyses

Data are expressed as the mean ± SD. Statistical differences were analyzed by Student’s test. The P value < 0.05 were considered statistically significant. And * denote P < 0.05, ** denote P < 0.01, *** denote P < 0.001, ns denote P > 0.05. All data were processed using GraphPad Prism 9.0 software.

## Results

### PIM-1 expression was upregulated in macrophages in the human OA synovium

**Fig. 1:**
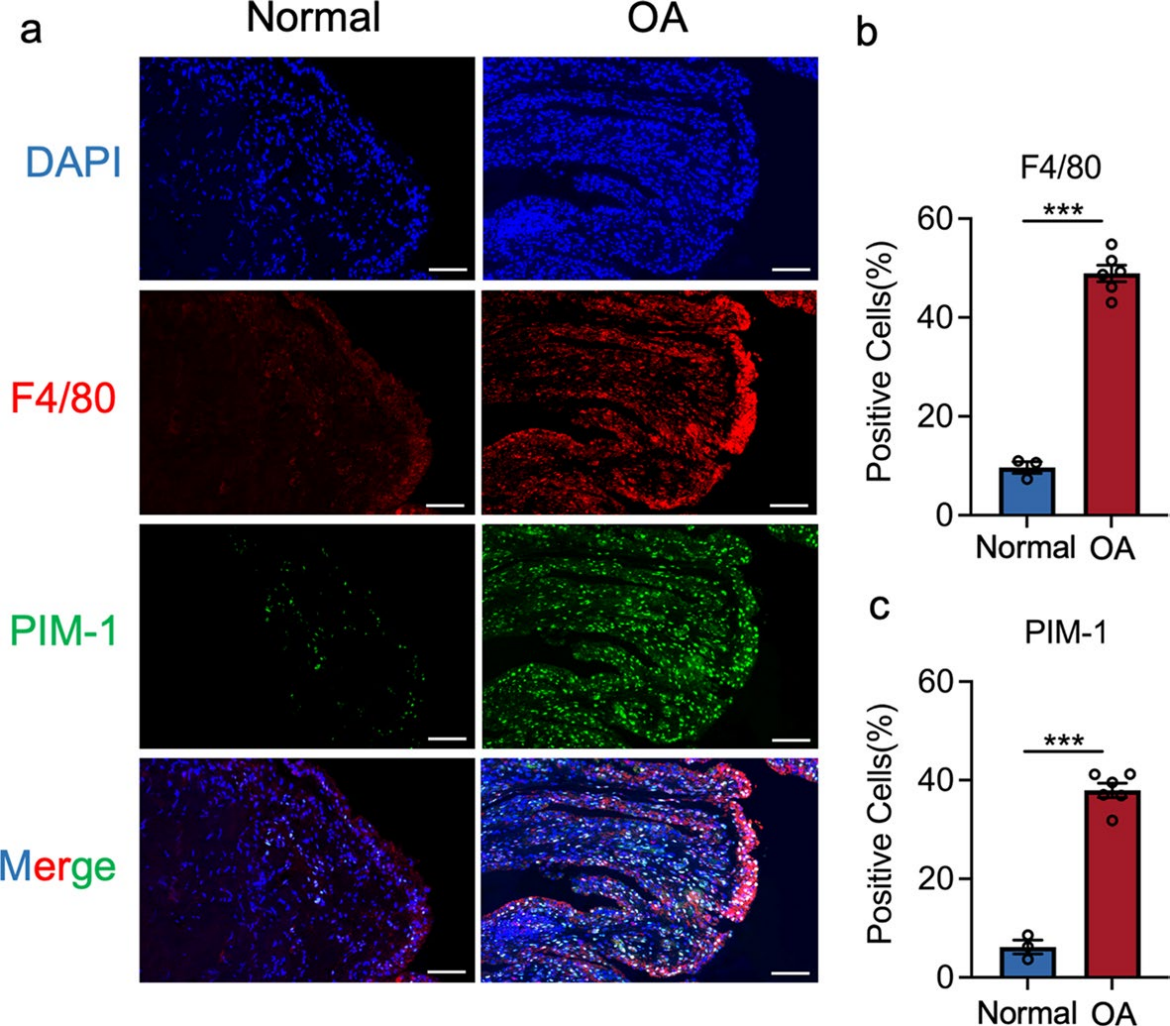
Macrophages and PIM-1 are upregulated in OA synovium. **a** Immunofluorescence images of F4/80 and PIM-1 in normal and OA human synovium. **b**, **c** Percentages of F4/80 and PIM-1-positive cells in human synovium. Statistics in **b** and **c** were performed using Student’s test. *P < 0.05, **P < 0.01, ***P < 0.001, ns P > 0.05. Bars represent mean ± SD. Scale bar = 100 μm

To explore the role of PIM-1 in macrophage and OA progression. We investigated the expression of macrophage marker (F4/80) in the human synovium. As in the previous study, the quantity of F4/80-positive cells was upregulated in OA humans in contrast to the normal synovium (Fig. 1a, b) [39]. In addition, the percentage of PIM-1-positive cells was also elevated in the synovium of OA humans (Fig. 1a, c). Intriguingly, PIM-1 is predominantly located in the macrophages, with colocalization of PIM-1 and F4/80. Taken together, these results indicated that macrophages accumulate in OA synovium with an increase in PIM-1 expression.

### Suppression of PIM-1 inhibited the NLRP3 inflammasome activation

**Fig. 2:**
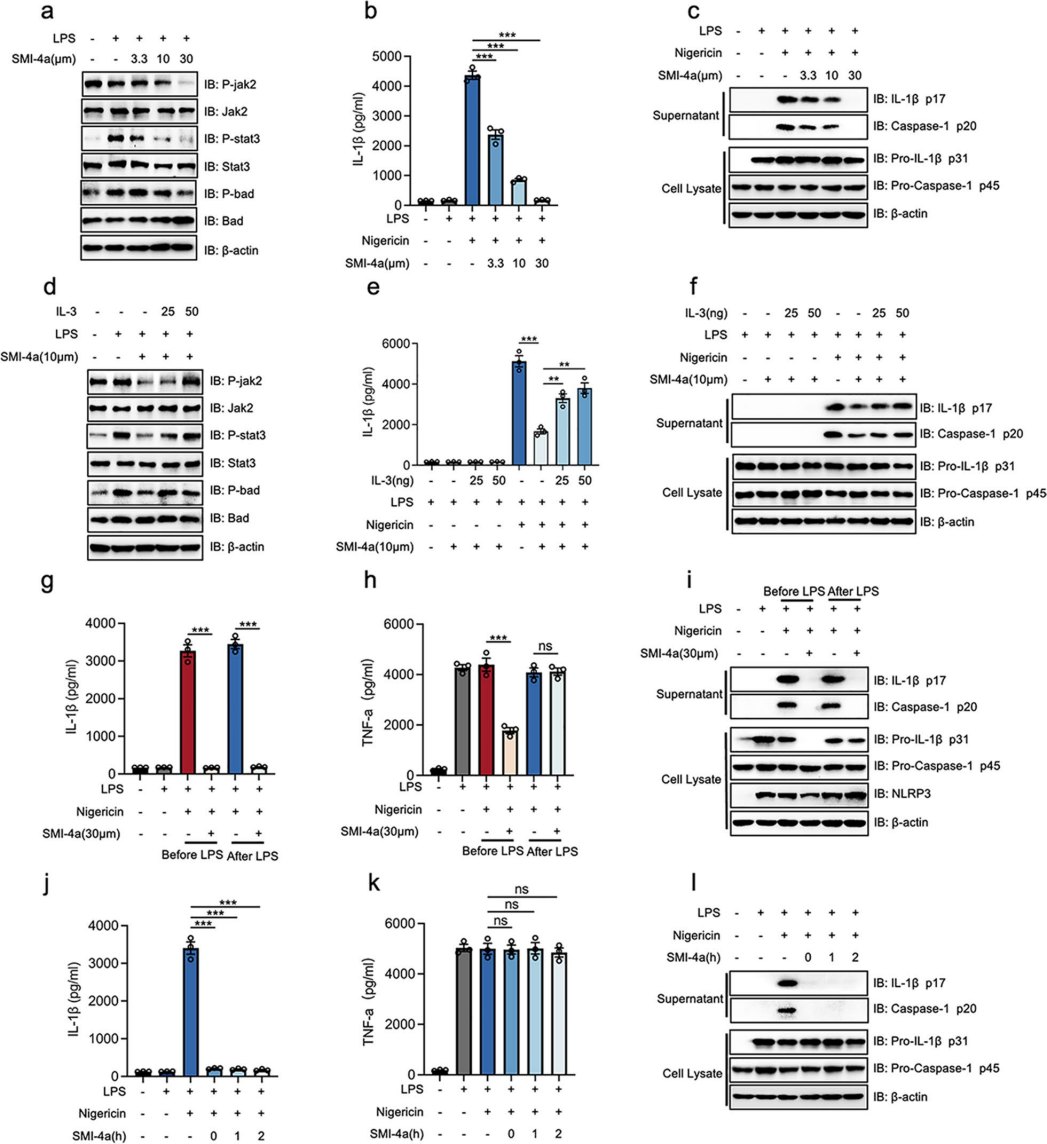
PIM-1 blockade inhibits the NLRP3 inflammasome activation in macrophages. **a** BMDMs were stimulated with LPS (1 µg/mL) for 4 h, whereafter co-incubated with SMI-4a in different doses for 2 h. The protein levels of cell extracts. **b**, **c** BMDMs were stimulated with LPS (1 µg/mL) for 4 h, whereafter co-incubated with SMI-4a in different doses for 2 h, followed by 45 min of nigericin stimulation. The secretion levels of IL-1β (**b**).The protein levels of supernatant and cell extracts (**c**). **d** LPS-primed BMDMs were treated with different doses of IL-3 for 2 h, whereafter co-incubated with SMI-4a (10 µM). The protein levels of cell extracts. **e**, **f** LPS-primed BMDMs were treated with different doses of IL-3 for 2 h, whereafter co-incubated with SMI-4a (10 µM), followed by 45 min of nigericin stimulation. The secretion levels of IL-1β (**e**). The protein levels of cell extracts (**f**). **g–i** BMDMs were co-incubated with SMI-4a (30 µM) for 2 h before or after 4 h LPS priming, followed by 45 min of nigericin stimulation. The secretion levels of IL-1β (**g**) and TNF-α (**h**). The protein levels of cell extracts (**i**). **j–l** BMDMs were stimulated with LPS (1 µg/mL) for 4 h, whereafter co-incubated with SMI-4a (30 µM) at different times, followed by 45 min of nigericin stimulation. The secretion levels of IL-1β (**j**) and TNF-α (**k**). The protein levels of supernatant and cell extracts (**l**). Statistics in **b**, **e**, **g**, **h**, **j**, and **k** were performed using Student’s test. *P < 0.05, **P < 0.01, ***P < 0.001, ns P > 0.05. Bars represent mean ± SD. N = 3 experiments

To confirm whether PIM-1 was specifically inhibited by SMI-4a in macrophages, we performed western blot analysis on LPS-primed BMDMs with SMI-4a for 2 h. The results showed that SMI-4 could downregulate the phosphorylation levels of jak2, stat3, and bad following LPS stimulation in a dose-dependent manner (3.3 μm,10 μm,30 μm) (Fig. 2a). This implies that PIM-1 activity was inhibited by SMI-4a. In addition, we cultured LPS-primed BMDMs with SMI-4a before nigericin or ATP stimulation. The results showed that SMI-4a dose-dependently (3.3 μm, 10 μm, 30 μm) inhibited the cleavage of pro-IL-1β (P31) and pro-caspase-1 (P45) into IL-1β (P17) and caspase-1 (P20) (Fig. 2b, c and Additional file 1: Fig. S1c, e) without altering TNF-a secretion (Additional file 1: Fig. S1a and d). IL-3, a growth factor, could upregulate the activity of PIM-1 through the JAK/STAT signaling way [41, 42]. Notably, our results showed that adding IL-3 (25 and 50ng/ml) could rescue the low phosphorylation levels of jak2, stat3, and bad induced by SMI-4a (10 μm) (Fig. 2d). This meant that PIM-1 activity was rescued by supplemental IL-3 (25 and 50ng/ml). Moreover, the activation of PIM-1 was capable of rescuing the downregulation of IL-1β secretion induced by SMI-4a (30 μm) (Fig. 2e and f) without altering TNF-a secretion (Additional file 1: Fig. S1b). These findings indicated that PIM-1 could participate in the assembly stage of the NLRP3 inflammasome activation without influencing the priming stage.

To observe the effect of PIM-1 on the priming stage and the assembly stage of the NLRP3 inflammasome in macrophages, SMI-4a was applied before and after LPS stimulation. The results showed that the production of TNF-α and the expression of pro-IL-1β and NLRP3 proteins were inhibited by the pretreatment of SMI-4a before LPS stimulation and these had no obvious change when SMI-4a treatment after LPS stimulation (Fig. 2g–i). These findings indicated that the suppression of PIM-1 could inhibit the NF-κB signal pathway and could inhibit NLRP3 inflammasome activation in the priming stage and the assembly stage. We stick to exploring the role of PIM-1 in the assembly stage when macrophages were treated with SMI-4a after LPS stimulation. Intriguingly, suppressed PIM-1 by SMI-4a showed a rapid inhibitory effect on NLRP3 inflammasome activation (Fig. 2j–l). To further eliminate the off-target possibility of SMI-4a, this study also found that another PIM-1 inhibitor, AZD1208, could rapidly inhibit the assembly stage of the NLRP3 inflammasome activation without influencing the priming stage (Additional file 1: Fig. S2).

### Blocking PIM-1 widely inhibits NLRP3, AIM2, NLRC4 inflammasome, and pyroptosis and specifically alleviated ASC oligomers form

**Fig. 3:**
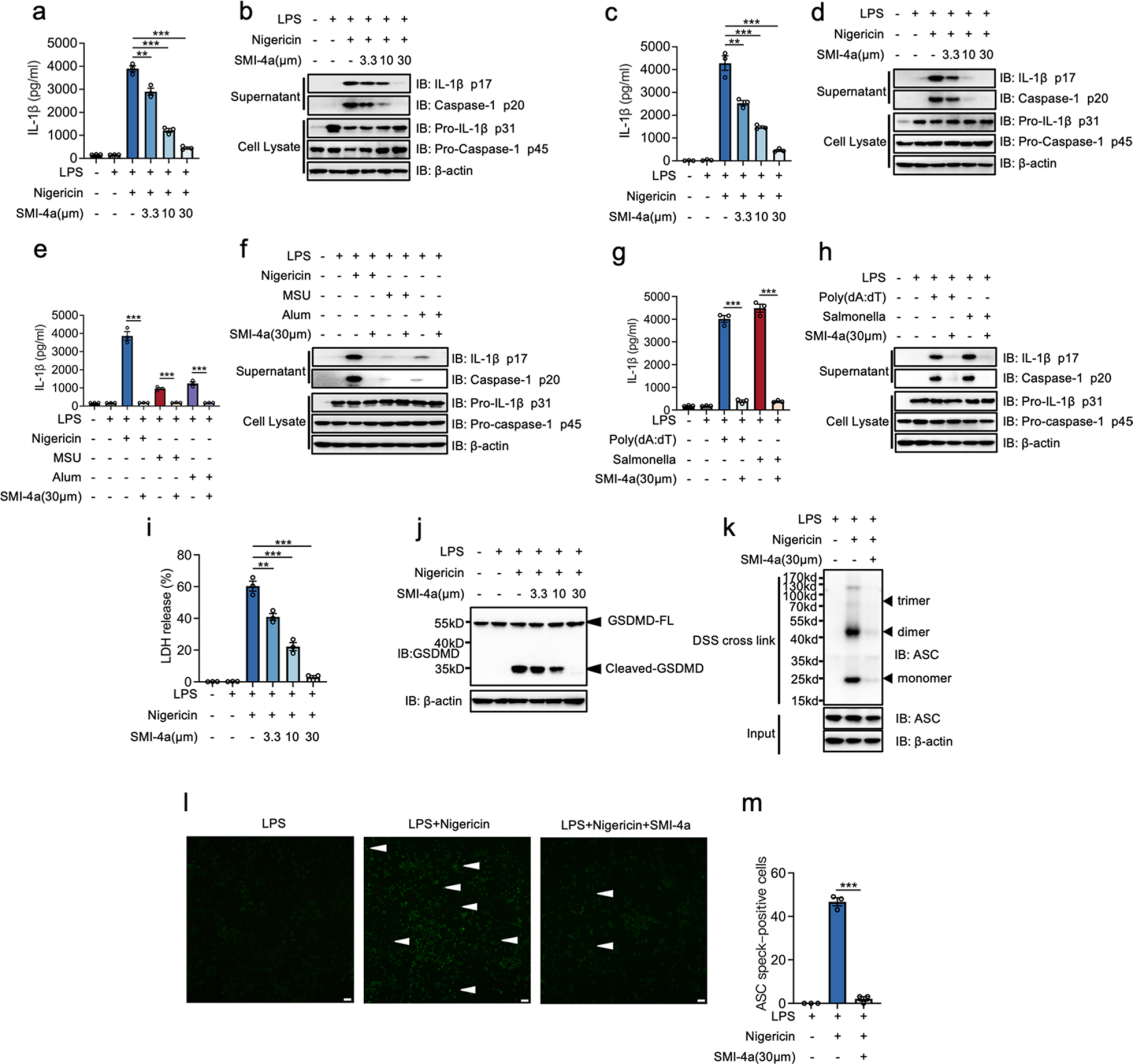
PIM-1 inhibition widely inhibits NLRP3, AIM2, NLRC4 inflammasome, and pyroptosis and specifically alleviated ASC oligomers form. **a–d** PMs from C57BL/6 mice **a**, **b** and THP-1 **c**, **d** were stimulated with LPS (1 µg/mL) for 4 h, whereafter co-incubated with SMI-4a, followed by 45 min of nigericin stimulation. The secretion levels of IL-1β (**a** and **c**). The protein levels of supernatant and cell extracts (**b** and **d**). **e**, **f** BMDMs were stimulated with LPS (1 µg/mL) for 4 h, whereafter co-incubated with SMI-4a (30 µM), and then challenged by nigericin for 45 min, MSU and Alum for 3 h. The secretion levels of IL-1β (**e**). The protein levels of supernatant and cell extracts (**f**). **g**, **h** BMDMs were stimulated with LPS (1 µg/mL) for 4 h, whereafter co-incubated with SMI-4a (30 µM) for 2 h, followed by handled with poly (dA:dT) transfection or Salmonella infection. The secretion levels of IL-1β (**g**). The protein levels of supernatant and cell extracts (**h**). **i–m** BMDMs were stimulated with LPS (1 µg/mL) for 4 h, whereafter co-incubated with SMI-4a, followed by 45 min of nigericin stimulation. The release level of LDH (**i**). The protein levels of supernatant and cell extracts (**j**). ASC oligomerization in cross-linked cytosolic pellets was analyzed by western blot (**k**). Representative immunofluorescence images (**l**) and quantification of ASC speck (**m**). Statistics in a, c, e, g, i, and m were performed using Student’s test. *P < 0.05, **P < 0.01, ***P < 0.001, ns P > 0.05. Bars represent mean ± SD. N = 3 experiments. Scale bar = 100 μm

To further investigated whether PIM-1 plays a common role in NLRP3 inflammasome activation, the function of PIM-1 on NLRP3 inflammasome activation was studied with various agonists in different macrophages. We found that SMI-4a dose-dependently inhibited the NLRP3 inflammasome activation in murine and human macrophages, such as PMs from male C57BL/6 mice (Fig. 3a, b, Additional file 1: Fig. S3a), THP-1 cells (Fig. 3c, d, Additional file 1: Fig. S3b), peripheral blood mononuclear cells (PBMCs) from healthy people (Additional file 1: Fig. S3c–e). Apart from nigericin, SMI-4a also could inhibit the caspase-1 cleavage and IL-1β secretion induced by MSU and Alum (Fig. 3e, f, Additional file 1: Fig. S3f).

To explore whether PIM-1 plays a specific role in the NLRP3 inflammasome activation, we investigated the role of PIM-1 in the NLRC4 and AIM2 inflammasome activation. The results revealed that SMI-4a had the inhibitory effect on AIM2 inflammasome and NLRC4 inflammasome, trigged by poly (dA:dT) transfection or Salmonella infection (Fig. 3g, h, Additional file 1: Fig. S3g), resulting in a decrease in IL-1β production.

LDH releasing and GSDMD cleavage are two pivotal characteristics of inflammasome induced-pyroptosis. Then, we found the suppression of PIM-1 by SMI-4a could inhibit releasing of LDH and block the cleavage of GSDMD (Fig. 3i, j, Additional file 1: Fig. S4a, b). Next, we studied how PIM-1 affects the assembly stage of the NLRP3 inflammasome formation. The interaction between NLRP3 and ASC was critical for the assembly of NLRP3 inflammasome. The co-immunoprecipitation (CO-IP) result indicated that SMI-4a did not influence the interaction of NLRP3 and ASC (Fig. S5). Upon the assembly of NLRP3 and ASC, subsequent ASC is recruited to form ASC oligomers and ASC specks. The result first indicated that PIM-1 suppression could specifically attenuate nigericin-induced DSS-crosslinked ASC oligomers and ASC-speck (Fig. 3k–m).

### Restraining of PIM-1 block Cl^-^ efflux to inhibit ASC oligomerization

**Fig. 4:**
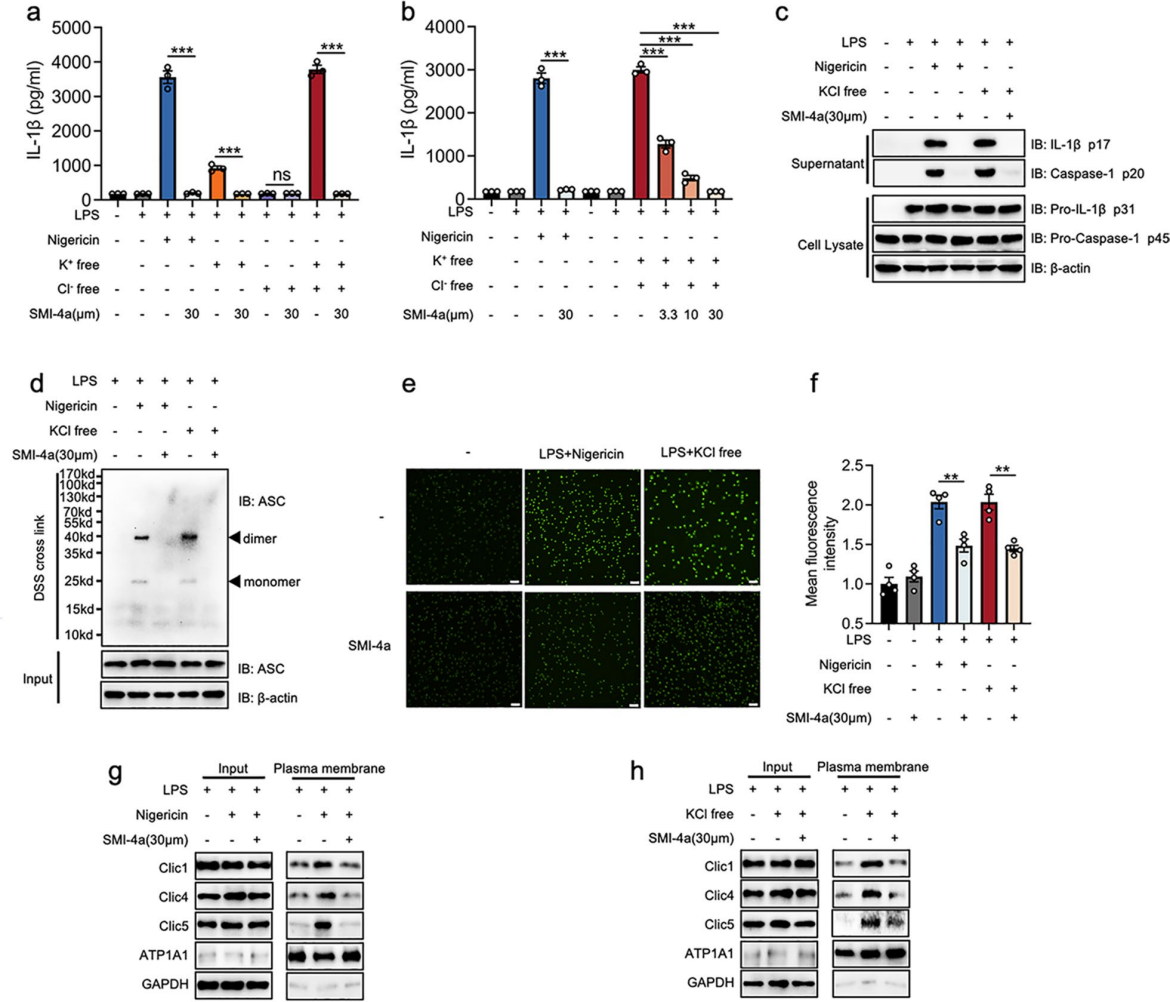
PIM-1 suppression blocks Cl^−^efflux to inhibit ASC oligomerization in macrophages. **a**–**c** LPS-primed BMDMs were treated with indicated doses of SMI-4a and then incubated in the indicated isotonic salt solution or incubated with nigericin for 45 min. **a**, **b**The secretion levels of IL-1β. **c** The protein levels of supernatant and cell extracts. **d** ASC oligomerization in cross-linked cytosolic pellets was analyzed by western blot. **e–h** LPS-primed BMDMs were treated with SMI-4a (30 µM) and then stimulated with nigericin for 15 min or incubated in K^+^ and Cl^−^ free solution for 30 min. **e**, **f** Representative fluorescence images of the intracellular Cl^–^ level were determined by MQAE **e** and the quantification of mean fluorescence intensity (**f**). **g**, **h** The indicated proteins in total lysates (input) and isolated plasma membrane of BMDMs were isolated to evaluate the translocation of CLICs by western blot. Statistics in **a**, **b** and **f** were performed using Student’s test. *P < 0.05, **P < 0.01, ***P < 0.001, ns P > 0.05. Bars represent mean ± SD. N = 3 experiments. Scale bar = 100 μm

Cl^−^ efflux plays a crucial role in ASC oligomerization and is an essential upstream event for NLRP3 inflammasome activation [38]. To understand the effect of PIM-1 on Cl^−^ efflux, we performed ion substitution experiments to drive the efflux of K^+^ or Cl^−^. Consistent with previous research, Cl^−^ free conditions alone did not cause IL-1β release, but it could further enhance IL-1β release caused by K^+^ free conditions [38]. Besides, no IL-1β was observed from these supernatants with the treatment of SMI-4a (Fig. 4a). Moreover, SMI-4a could dose-dependently inhibit the release of IL-1β in the K^+^ and Cl^−^ free conditions (Fig. 4b,  c). Consistent with previous research, we found K^+^ and Cl^−^ free conditions could activate the ASC oligomerization [38]. SMI-4a could alleviate ASC oligomerization caused by nigericin or K^+^ and Cl^−^ free conditions (Fig. 4d). Together, these results revealed that the PIM-1 suppression inhibited NLRP3 inflammasome activation might be related to Cl^−^ efflux.

As shown in Fig. 4e, f, nigericin or K^+^ and Cl^−^ free condition markedly increased the intracellular fluorescence intensity, indicating the decrease of intracellular Cl^−^ levels and the enhancement of Cl^−^ efflux. However, these processes were blocked by SMI-4a. The chloride intracellular channel proteins (CLICs) family, comprised of CLIC1, CLIC4, and CLIC5, were present in both the cytosol and plasma membranes. CLICs could translocate to the plasma membrane, forming anion channels to mediate intracellular Cl^−^ efflux [34, 43]. We further sought to explore whether SMI-4a-induced Cl^−^ efflux change is mediated by the blockade of CLICs translocation. Western blot analysis indicated that SMI-4a could block the CLICs translocation induced by nigericin and K^+^ and Cl^−^ free conditions (Fig. 4g, h). Taken together, we first indicated that PIM-1 played a critical role in CLICs translocation and Cl^−^ efflux to achieve the ASC oligomerization and NLRP3 inflammasome activation.

### Suppression of PIM-1 block Cl^-^ efflux via mitochondrial ROS

**Fig. 5:**
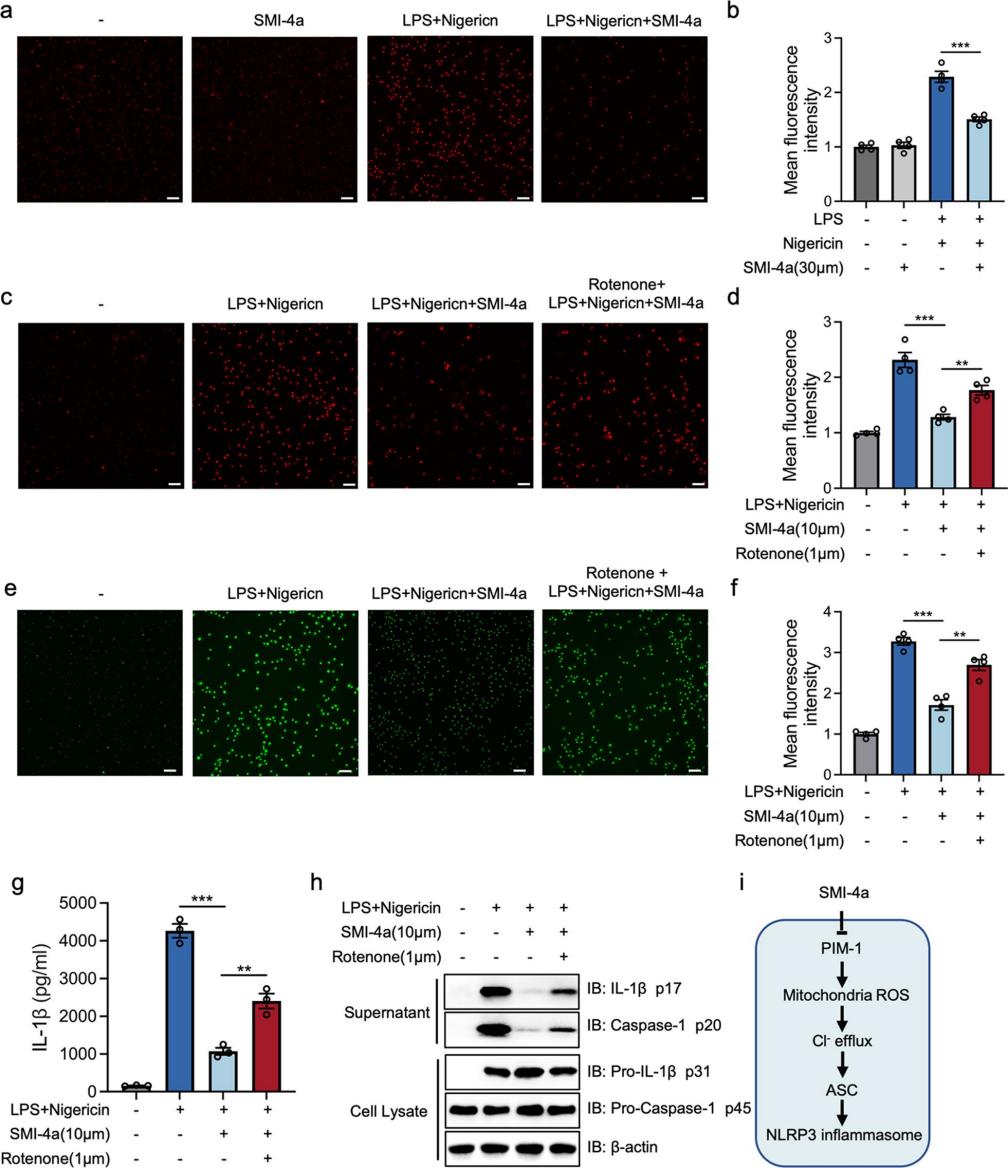
PIM-1 suppression inhibits the production of mitochondrial ROS to block Cl- efflux in macrophages. **a**, **b** BMDMs were stimulated with LPS (1 µg/mL) for 4 h, whereafter co-incubated with SMI-4a and nigericin stimulation for 45 min. Representative fluorescence images of the intracellular mitochondrial ROS were determined by MitoSOX Red probe (**a**) and the quantification of mean fluorescence intensity (**b**). **c–h** BMDMs were stimulated with LPS (1 µg/mL) for 4 h, whereafter co-incubated with rotenone (1 μm) for 2 h, followed by 45 min of SMI-4a (10 μm) and nigericin. Representative fluorescence images of the intracellular mitochondrial ROS were determined by MitoSOX Red probe (**c**) and the quantification of mean fluorescence intensity (**d**). Representative fluorescence images of the intracellular Cl^−^ level were determined by MQAE (**e**) and the quantification of mean fluorescence intensity (**f**). The secretion levels of IL-1β (**g**). The protein levels of supernatant and cell extracts (**h**). **i** Model for the mechanism of PIM-1 in the assembly stage of NLRP3 inflammasome activation. Statistics in **b**, **d**, **f**, and **g** were performed using Student’s test. *P < 0.05, **P < 0.01, ***P < 0.001, ns P > 0.05. Bars represent mean ± SD. N = 4 experiments. Scale bar = 100 μm

Mitochondrial ROS act upstream of Cl^−^ efflux to accelerate NLRP3 inflammasome activation and plays a crucial role in the activation of AIM2, and NLRC4 inflammasome [34, 44, 45]. To investigate whether PIM-1 suppression blocked Cl^−^ efflux and NLRP3 inflammasome activation through the regulation of mitochondrial ROS, we first used the MitoSOX Red probe to detect the level of mitochondrial ROS in macrophages. The result revealed that the NLRP3 inflammation activation accompanied by the production of mitochondrial ROS and SMI-4a could inhibit the levels of mitochondrial ROS (Fig. 5a, b). Moreover, the production of mitochondrial ROS was significantly increased when LPS-primed BMDMs were treated with rotenone, an mitochondrial electron transport chain complex I inhibitor, before the incubation of SMI-4a and nigericin. This finding indicated that rotenone blocked the inhibition function of SMI-4a toward mitochondrial ROS production (Fig. 5c, d). In addition, the activation of Cl^−^ efflux and the NLRP3 inflammasome were enhanced after the pretreatment of rotenone (Fig. 5e–h). To sum up, these results first suggested that PIM-1 inhibition restrained the production of mitochondrial ROS and blocked Cl^−^ efflux, resulting in the repressive effects on NLRP3 inflammasome.

### PIM-1 inhibition suppress chondrocytes apoptosis and improve chondrocytes proliferation and ECM metabolism in the co-culture system

**Fig. 6:**
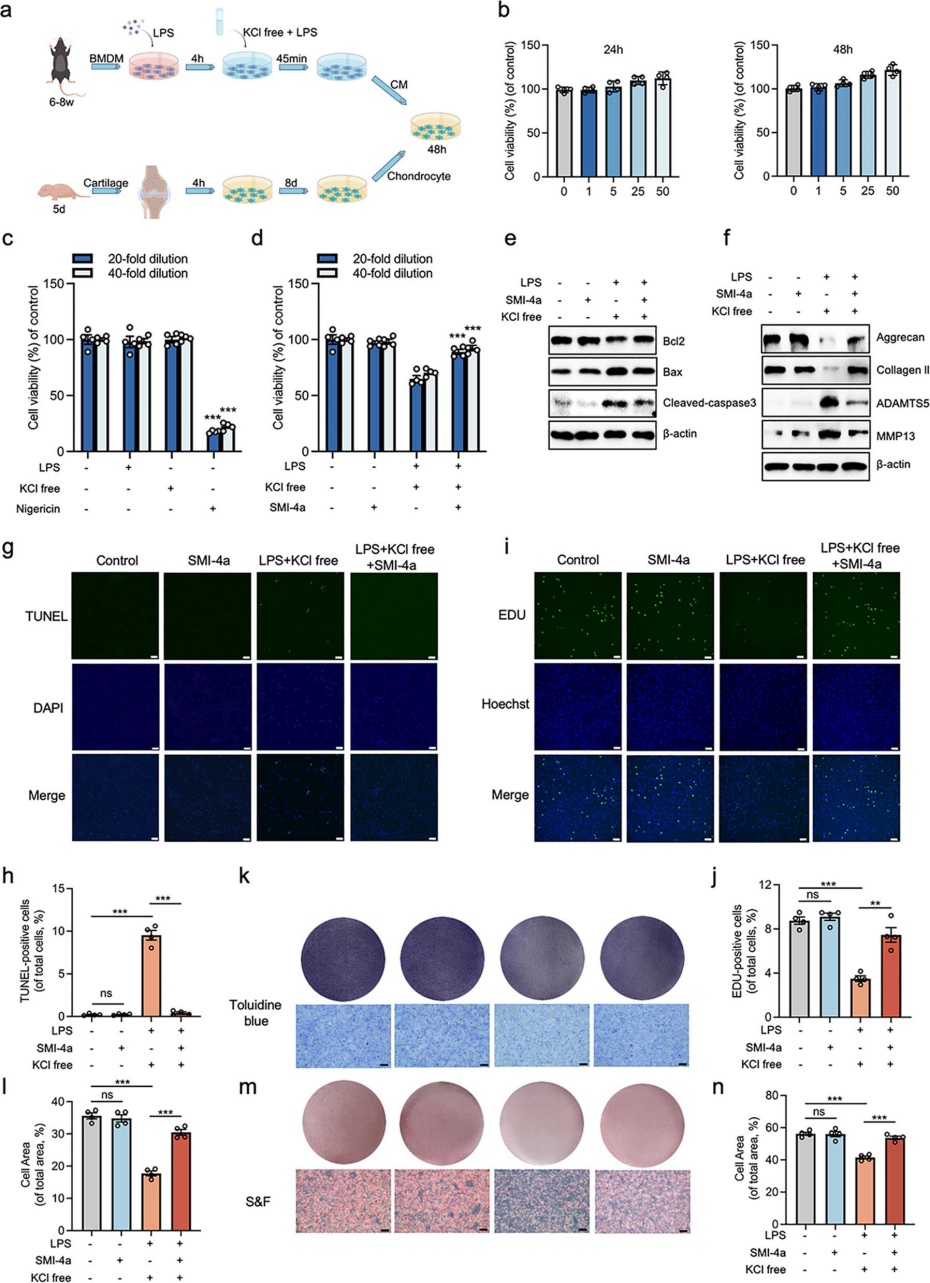
PIM-1 suppression prevents chondrocyte damage in the co-culture system. **a** Macrophages and chondrocytes co-culture system establishment model diagram. (Created by Figdraw). **b** The cytotoxic effect of SMI-4a on the chondrocytes was evaluated at various concentrations for 24 and 48 h by CCK8 assay. **c**, **d** For the co-culture system, chondrocytes were cultured for 48 h in 20 or 40-fold dilution of macrophage CM, and the cytotoxic effect on chondrocytes was evaluated by CCK8 assay. **e** The protein levels of Bcl-2, cleaved-caspase3, and Bax of chondrocytes cultured for 48 h were detected by western blot. **f** The protein levels of Aggrecan, Collagen II, ADAMTS5, and MMP13 of chondrocytes cultured for 48 h were detected by western blot. **g**, **h** The apoptotic levels of chondrocytes cultured for 48 h were determined by TUNEL staining. **i**, **j** The proliferation levels of chondrocytes cultured for 48 h were determined by EDU staining. Toluidine blue **k**, **l** and S&F **m**, **n** staining of chondrocytes cultured for 48 h. Statistics in **b**, **c**, **d**, **h**, **j**, **l**, and **n** were performed using Student’s test. *P < 0.05, **P < 0.01, ***P < 0.001, ns P > 0.05. Bars represent mean ± SD. N = 4 experiments. Scale bar = 100 μm

We further utilized the co-culture system to investigate the interaction of macrophages and chondrocytes [46]. First, we should detect the cytotoxic effects of SMI-4a on chondrocytes. The results showed that SMI-4a concentration < 50 µM has no noticeable cytotoxic effects on chondrocytes treated within 24 or 48 h (Fig. 6b). Subsequently, we stimulated macrophages with various single ingredients involved in NLPR3 inflammasome activation and cultured chondrocytes with CM. Intriguingly, the viability of chondrocytes was decreased in the Nigericin group compared with the Control group, whereas other groups had no obvious change. This implied that the CM collected from macrophages treated with nigericin had apparent toxic effects on chondrocytes (Fig. 6c). Ultimately, we gave up nigericin and selected K^+^ and Cl^-^ free conditions to activate the NLRP3 inflammasome. Therefore, a modified macrophage-chondrocyte co-culture system was established (Fig. 6a).

In this co-culture system, the viability of chondrocytes was increased under the treatment of SMI-4a compared with that of the LPS + KCl free group (Fig. 6d). Consistent results were observed from the TUNEL staining (Fig. 6g, h). Moreover, the expression of cleaved-caspase3 and Bax was decreased, and the expression of Bcl-2 was increased with the treatment of SMI-4a compared with those in the LPS + KCl free group (Fig. 6e). These findings indicated that PIM-1 suppression could alleviate chondrocyte apoptosis. In addition, the expression levels of collagen II and aggrecan were increased, and the expression levels of MMP13 and ADAMTS5 were decreased under the treatments with SMI-4a compared with those in the LPS + KCl free group (Fig. 6f). These results manifested that PIM-1 suppression could restore the ECM metabolic of chondrocytes. EDU staining indicated that the proliferation levels of chondrocytes were increased with the treatment of SMI-4a compared with that of the LPS + KCl free group (Fig. 6i and j). Based on the results, the number of chondrocytes was increased with the treatment of SMI-4a, as shown by the increase in staining intensity and cellular area fraction of toluidine blue and S&F staining (Fig. 6k–n). Those results first demonstrated that PIM-1 suppression had a beneficial effect on chondrocyte proliferation.

### PIM-1 inhibition could ameliorate the progression of OA in mice DMM model

**Fig. 7:**
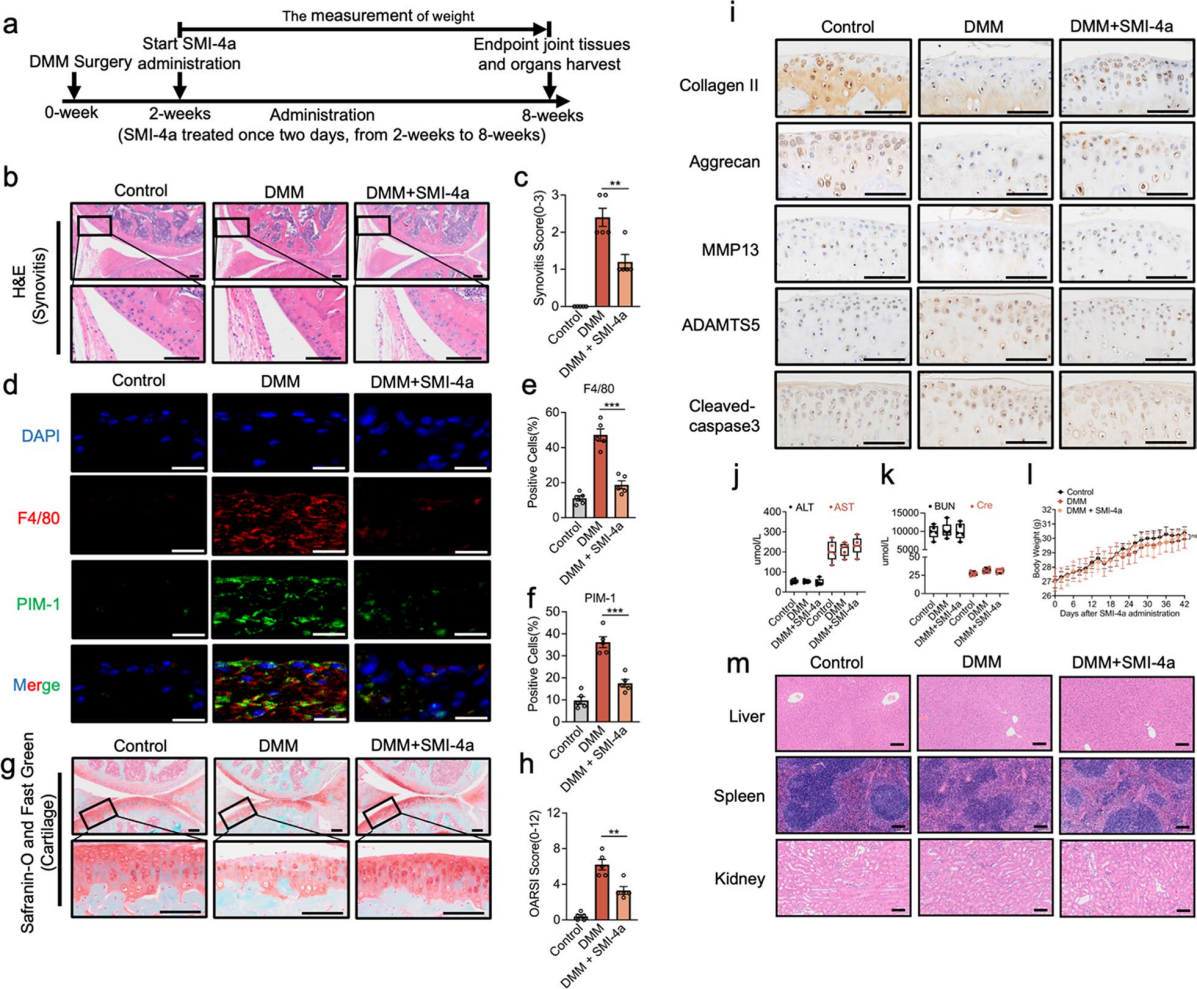
PIM-1 inhibition amelioratse the progression of OA in mice DMM model. **a** Timeline of SMI-4a intervention experiment in the DMM model. **b** Representative images of Haematoxylin and eosin (H&E) staining of the synovium in three groups at 8 weeks post-surgery. **c** The scores of synovitis in three groups. **d** Immunofluorescence images of F4/80 and PIM-1 in three groups at 8 weeks post-surgery. **e**, **f** Percentages of F4/80 **e** and PIM-1 **f** -positive cells in three groups at 8 weeks post-surgery. **g** Representative images of S&F staining of cartilage in three groups at 8 weeks post-surgery. **h** The OARIS scores of cartilage in three groups. **i** Representative images of immunohistochemical of Collagen II, Aggrecan, MMP13, ADAMTS5, and cleaved-caspase3 in three groups of mice cartilage. **j**, **k** The assessment of serum levels of ALT, AST, BUN, and Cre in three groups. **l** The measurement of body weight of mice in three groups. **m** Representative images of H&E staining of the liver, spleen, and kidney in three groups. Statistics in **c**, **e**, **f**, **h**, **j**, **k**, and **l** were performed using Student’s test. *P < 0.05, **P < 0.01, ***P < 0.001, ns P > 0.05. Bars represent mean ± SD. N = 5 mice from each group. Scale bar, 100 μm for **b**, **g**, **i**, **m** and 20 μm for **d**

The study further explored the potential treatment effect of PIM-1 inhibition on OA in vivo. The H&E staining showed that DMM mice treatment with SMI-4a ameliorated the hypertrophy and hyperplasia of the synovium and reduced the thickness of the synovial lining cell layer (Fig. 7b). Results indicated that SMI-4a could reduce synovitis scores, which were elevated in the DMM group (Fig. 7c). Besides, in line with human OA synovium, PIM-1 colocalized with macrophages (F4/80) was significant upregulation in the DMM mice (Fig. 7d–f). However, inhibition of PIM-1 by treatment with SMI-4a showed that these positive cells in synovial membrane were prominent downregulation. In addition, the S&F staining showed that the treatment of SMI-4a ameliorated the rough and erosive articular surface cartilage, chondrocytes reduction, and the abnormally narrow joint space compared with the DMM group. Results indicated that SMI-4a could reduce OARSI scores, which were elevated in the DMM group (Fig. 7h). Immunohistochemistry of these proteins plays a characteristic pathological indicative role in the development of OA, such as collagen II, aggrecan, MMP13, ADAMTS5, and cleaved-caspase3. Compared with the DMM group, treatment with SMI-4a ameliorated the downregulation of collagen II and aggrecan and restrained the ascent of MMP13, ADAMTS5, and cleaved-caspase3 (Fig. 7i). For safety concerns of SMI-4a, the serum levels of liver enzymes alanine transaminase (ALT) and aspartate aminotransferase (AST) were tested for hepatotoxicity (Fig. 7j), blood urea nitrogen (BUN) and creatinine (Cre) were evaluated for nephrotoxicity (Fig. 7k), and the body weight was assessed for the systemic toxicities (Fig. 7l). Compared with the Control and DMM group, all these parameters were not changed obviously after the SMI-4a treatment. Moreover, no noticeable change was observed from the H&E staining of the liver, spleen, and kidney (Fig. 7m). Our in vivo results first indicated that PIM-1 inhibition could play a protective role in OA mice.

## Discussion

Synovitis and cartilage destruction are the main clinical manifestations and could occur at any stage of osteoarthritis. Several arthritis risky factors, such as metabolites, crystals, and mitochondrial dysfunction, can mediate the NLRP3 inflammasome activation and pyroptosis of synovial macrophages [47]. Synovial macrophages secret IL-1β to aggravate synovial inflammation and further damage cartilage in OA [47]. Over the years, therapeutics targeting NLRP3 inflammasome of macrophages were largely effective during OA treatment [48]. However, no targets could effectively slow and cure the progression of OA. Here, we demonstrated PIM-1 was a possible therapeutic target for OA that regulated the activation of NLRP3 inflammasome in macrophages. We showed that PIM-1 expression had a significant upregulation in macrophage in the human OA synovium. PIM-1 suppression could rapidly inhibit the NLRP3 inflammasome activation in the assembly stage. Moreover, it had broad inhibitory functions against NLRP3, NLRC4, and AIM2 inflammasome activation and pyroptotic cell death. Furthermore, we unveiled that PIM-1 inhibition specifically blocked the ASC oligomerization and NLRP3 inflammasome activation via the mitochondrial ROS/Cl^−^ efflux pathway. Additionally, PIM-1 suppression showed chondroprotective effects in a modified co-culture system. Finally, PIM-1 inhibition ameliorated synovitis and cartilage damage in the mice OA model. Thus, PIM-1 may be a promising target candidate for macrophage and will contribute to the therapy of OA.

PIM-1, as a downstream effector of many cytokine signaling pathways, has been affirmed that could facilitate inflammation responses in macrophages. Of note, the number of macrophages and inflammatory factor expression increased in the synovium of OA[30]. Previous studies indicated that PIM-1 in macrophages plays an important role in the progression of inflammatory diseases. Here, we first found that PIM-1 expression was increased in macrophages in the human OA synovium. PIM-1 could modulate the NLRP3 inflammasome activation through the Pim-1/NFATc1/NLRP3 pathway or TLR4-NF-κB-NLRP3 Pathway [24, 37]. However, whether PIM-1 participates in the assembly stage of NLRP3 inflammasome activation remains unclear. Moreover, the joint cavity presents various DAMPs that initiated NLRP3 inflammasome activation in the assembly stage. Therefore, there was crucial for us to understand the role of PIM-1 in the assembly stage. In vitro study, we demonstrated that PIM-1 inhibition by specific inhibitors produced a dose-dependent inhibition effect on NLRP3 inflammasome activation (Fig. 2b, c, additional file 1: Fig. S1c and e and S2a, c, d and f). Moreover, the restoration of PIM-1 activity was accompanied by significant upregulation of the IL-1β secretion (Fig. 2d–f). Intriguingly, these processes could not influence the priming stage when PIM-1 specific inhibitor was treated after LPS stimulation (Additional file 1: Figs. S1a, and d and S2b and e). Therefore, this study first demonstrated that PIM-1 had a crucial function in the assembly stage of NLRP3 inflammasome in macrophages. Intriguing, previous studies show that the inhibition of PIM-1 was accompanied by the downregulation of NLRP3 protein [24, 36, 37]. PIM-1 knockout leads to the decreased expression of pro-IL-1β protein [24]. These same processes also took place in the macrophages when PIM-1 specific inhibitor was treated before LPS stimulation (Fig. 2i). However, the NLRP3 and pro-IL-1β protein expression were not appreciably changed when macrophages were treated with PIM-1 specific inhibitor after LPS stimulation. These results indicated PIM-1 had multiple functions on both the primed stage and assembly stage of NLRP3 inflammasome. These different phenomena might be associated with treatment time and mechanisms of action in this experiment. Moreover, GSDMED-mediated pyroptosis was considered as the downstream of NLRP3 inflammasome during the inflammation reactions. In the present study, we also demonstrated that PIM-1 suppression rapidly inhibited the cleaving of GSDMED and pyroptosis in macrophages (Fig. 3i, j and Additional file 1: Figs. S4a, b). Therefore, these findings first suggested that PIM-1 contributes to NLRP3 inflammasome activation and pyroptosis in macrophages via the influence of the assembly stage.

This study found that PIM-1 suppression could specifically block ASC oligomerization in the assembly stage. Recent studies have proved that Cl^−^ efflux acts upstream of the ASC oligomerization and inhibition of Cl^−^ efflux currents reduces NLRP3 inflammasome activation [34]. Here, our study demonstrated that PIM-1 suppression could inhibit ASC oligomerization by the blockade of Cl^−^ efflux, which is induced by the transformation of CLICs (Fig. 4). Intriguingly, Ticagrelor can suppress Cl^−^ efflux -induced NLRP3 inflammasome activation through the degradation of CLICs, but it has no substantial inhibitory function on the NLRC4 inflammasome activation or pyroptosis [43]. Mitochondrial ROS acts upstream of Cl^−^ efflux and is considered as commonly upstream of NLRP3, AIM2, NLRC4 inflammasome, and pyroptosis. Besides, PIM-1 plays a regulatory role in mitochondrial integrity, which is related to the production of mitochondrial ROS [49, 50]. Therefore, PIM-1 may participate in NLRP3 inflammasome activation and pyroptosis by mediating mitochondrial ROS production. In this study, we indicated that PIM-1 suppression decreased the levels of mitochondrial ROS induced by the medication of LPS and nigericin (Fig. 5a, b). In addition, the use of a mitochondrial ROS activator could rescue the alleviation effect of PIM-1 specific inhibitor on Cl^−^ efflux and NLRP3 inflammasome activation (Fig. 5c–h). Therefore, these results first indicated that PIM-1 suppression was capable of blocking mitochondrial ROS/Cl^−^ efflux signaling way, thus inhibiting NLRP3 inflammasome activation (Fig. 5i). This might explain the broad suppression effect of PIM-1 inhibition in these inflammasome activations.

**Fig. 8:**
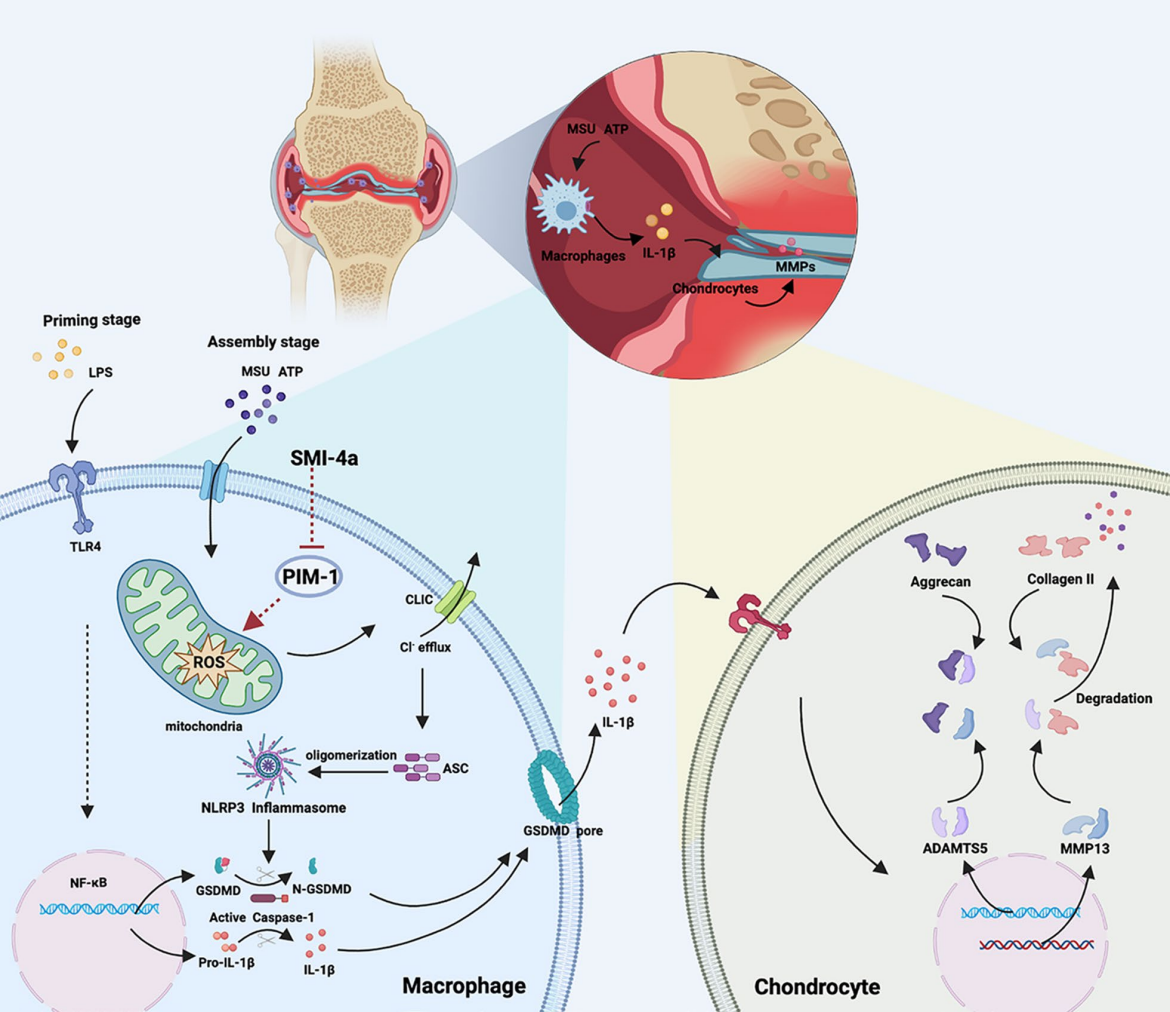
Schematic diagram of targeting macrophagic PIM-1 resulting in the potential chondroprotective effect against osteoarthritis. The NLRP3 inflammasome of macrophages was activated by risk factors in the synovial microenvironment. Suppression PIM-1 was capable of blocking Mitochondrial ROS/Cl^−^ efflux signaling pathway, thus inhibiting NLRP3 inflammasome activation and IL-1β secretion. Eventually, the stimulation on chondrocytes by IL-1β was reduced, resulting in chondroprotection against OA. (Created with BioRender.com)

PIM-1 suppression inhibited NLRP3 inflammasome activation in macrophages resulting in chondroprotective effects (Fig. 6). The interaction between macrophages and chondrocytes was crucial in the OA [51, 52]. The level of IL-1β is elevated in the synovial fluid of OA [53, 54]. IL-1β breaks the balance of catabolism and anabolism of chondrocytes and increases the activity of ECM degradation-related proteases (MMP13 and ADAMTS5), which are considered as common mediators of cartilage destruction in OA [1, 46, 55]. It is reported that blocking the interaction of IL-1β and chondrocytes produces a strong chondroprotective effect [56]. We established a modified co-culture system to distinguish the validity of SMI-4a on chondrocytes by inhibiting IL-1β secretion(Fig. 6a). Finally, our results suggested that PIM-1 suppression had chondroprotective effects by mediating the interaction of macrophages and chondrocytes (Fig. 6). It is worth mentioning that K^+^ and Cl^−^ free condition was chosen to activate the NLRP3 inflammasome because it had no toxic side effects on chondrocytes (Fig. 6c). Moreover, metabolites presented in the CM were produced only at the NLRP3 inflammasome assembly stage due to the reperfusion of K^+^ and Cl^−^ free solution. In vivo experiments, after therapy with SMI-4a (a specific inhibitor of PIM-1), the expression of PIM-1 in the synovium of DMM-model was significantly downregulated. Moreover, PIM-1 suppression showed protection function against synovial hyperplasia and cartilage damage (Fig. 7). Together, this was first demonstrated that PIM-1 may be a promising candidate target for the therapy of OA (Fig. 8).

The current study still had the following limitations that should be considered. First, the pathological progression of OA involves many compartments, but this research only explored the interaction of macrophages and chondrocytes. Second, articular injection of SMI-4a could target the synovial macrophage directly. However, local infections and suppurative arthritis might be caused by repeated intra-articular injections. Therefore, more efficient delivery methods still need to be explored. Third, we committed to exploring the chondroprotective effects by inhibiting PIM-1 in macrophages. Whether PIM-1 suppression directly exerts chondroprotective effects is unknown, which needs further study.

In summary, our study highlighted that PIM-1 was an effective target for the treatment of OA. PIM-1 suppression could inhibit NLRP3 inflammasome and GSDMED-mediated pyroptosis via blocking mitochondrial ROS/Cl^−^ efflux in the assembly stage. Our study showed that PIM-1 specific inhibitor could effectively treat OA in mice. Therefore, PIM-1 represented a new class of promising targets as a treatment of OA to target these mechanisms in macrophages and widened the road to therapeutic strategies for OA.

## Supplementary Information

**Additional file 1. Fig. S1.** PIM-1 blockade inhibites the NLRP3 inflammasome activation in macrophages. **Fig. S2.** PIM-1 inhibitor AZD1208 inhibites the NLRP3 inflammasome activation in macrophages. **Fig. S3.** SMI-4a suppresses the NLRP3 inflammasome activation in macrophages. **Fig. S4.** SMI-4a inhibits the maturity of Cleaved-GSDMD in macrophages.** Fig. S5.** SMI-4a has no effect on the interaction of NLRP3 and ASC in macrophages.

## Data Availability

All the data used or/and analyzed in this study are available from the corresponding author upon reasonable request.

## References

[1] Pap T, Korb-Pap A (2015). Cartilage damage in osteoarthritis and rheumatoid arthritis–two unequal siblings. Nat Rev Rheumatol.

[2] Pattison LA (2021). Cell-cell interactions in joint pain: rheumatoid arthritis and osteoarthritis. Pain.

[3] Kraus VB (2016). Direct in vivo evidence of activated macrophages in human osteoarthritis. Osteoarthritis Cartilage.

[4] Zeisbrich M (2018). Hypermetabolic macrophages in rheumatoid arthritis and coronary artery disease due to glycogen synthase kinase 3b inactivation. Ann Rheum Dis.

[5] Hughes MM, O’Neill LAJ (2018). Metabolic regulation of NLRP3. Immunol Rev.

[6] He Y, Hara H, Núñez G (2016). Mechanism and regulation of NLRP3 inflammasome activation. Trends Biochem Sci.

[7] Clavijo-Cornejo D (2016). The overexpression of NALP3 inflammasome in knee osteoarthritis is associated with synovial membrane prolidase and NADPH Oxidase 2. Oxid Med Cell Longev.

[8] Guo C (2018). NLRP3 inflammasome activation contributes to the pathogenesis of rheumatoid arthritis. Clin Exp Immunol.

[9] McAllister MJ (2018). NLRP3 as a potentially novel biomarker for the management of osteoarthritis. Osteoarthritis Cartilage.

[10] Dinarello CA, Simon A, van der Meer JW (2012). Treating inflammation by blocking interleukin-1 in a broad spectrum of diseases. Nat Rev Drug Discov.

[11] He WT (2015). Gasdermin D is an executor of pyroptosis and required for interleukin-1β secretion. Cell Res.

[12] Afonina IS (2017). Limiting inflammation-the negative regulation of NF-κB and the NLRP3 inflammasome. Nat Immunol.

[13] Swanson KV, Deng M, Ting JP (2019). The NLRP3 inflammasome: molecular activation and regulation to therapeutics. Nat Rev Immunol.

[14] Mangan MSJ (2018). Targeting the NLRP3 inflammasome in inflammatory diseases. Nat Rev Drug Discov.

[15] Xu J, Núñez G (2023). The NLRP3 inflammasome: activation and regulation. Trends Biochem Sci.

[16] Shi J (2015). Cleavage of GSDMD by inflammatory caspases determines pyroptotic cell death. Nature.

[17] Wang K (2020). Structural mechanism for GSDMD Targeting by Autoprocessed Caspases in pyroptosis. Cell.

[18] Lambert C (2020). The Damage-Associated molecular patterns (DAMPs) as potential targets to treat osteoarthritis: perspectives from a review of the literature. Front Med (Lausanne).

[19] Millerand M, Berenbaum F, Jacques C (2019). Danger signals and inflammaging in osteoarthritis. Clin Exp Rheumatol.

[20] Luszczak S (2020). PIM kinase inhibition: co-targeted therapeutic approaches in prostate cancer. Signal Transduct Target Ther.

[21] Amaravadi R, Thompson CB (2005). The survival kinases akt and pim as potential pharmacological targets. J Clin Invest.

[22] Ha YJ (2019). PIM-1 kinase is a novel regulator of proinflammatory cytokine-mediated responses in rheumatoid arthritis fibroblast-like synoviocytes. Rheumatology (Oxford).

[23] Lilly M (1992). Sustained expression of the pim-1 kinase is specifically induced in myeloid cells by cytokines whose receptors are structurally related. Oncogene.

[24] Baek HS (2020). Anti-inflammatory Effects of the novel PIM kinase inhibitor KMU-470 in RAW 264.7 cells through the TLR4-NF-κB-NLRP3 pathway. Int J Mol Sci.

[25] Kim KT, Levis M, Small D (2006). Constitutively activated FLT3 phosphorylates BAD partially through pim-1. Br J Haematol.

[26] Paíno T (2017). The novel Pan-PIM kinase inhibitor, PIM447, displays dual antimyeloma and bone-protective effects, and potently synergizes with current standards of Care. Clin Cancer Res.

[27] Maney NJ (2021). Pim Kinases as therapeutic targets in early rheumatoid arthritis. Arthritis Rheumatol.

[28] Zhang X (2020). Inhibition of PIM1 kinase attenuates bleomycin-induced pulmonary fibrosis in mice by modulating the ZEB1/E-cadherin pathway in alveolar epithelial cells. Mol Immunol.

[29] Cao Y (2021). PIM1 inhibition attenuated endotoxin-induced acute lung injury through modulating ELK3/ICAM1 axis on pulmonary microvascular endothelial cells. Inflamm Res.

[30] Kapoor M (2011). Role of proinflammatory cytokines in the pathophysiology of osteoarthritis. Nat Rev Rheumatol.

[31] Busso N, So A (2012). Microcrystals as DAMPs and their role in joint inflammation. Rheumatology (Oxford).

[32] Martinon F (2006). Gout-associated uric acid crystals activate the NALP3 inflammasome. Nature.

[33] Dominic A, Le NT, Takahashi M (2022). Loop between NLRP3 inflammasome and reactive oxygen species. Antioxid Redox Signal.

[34] Tang T (2017). CLICs-dependent chloride efflux is an essential and proximal upstream event for NLRP3 inflammasome activation. Nat Commun.

[35] Chauhan SS (2020). PIM kinases alter mitochondrial dynamics and chemosensitivity in lung cancer. Oncogene.

[36] Wang X (2022). Inhibition of CEBPB attenuates Lupus Nephritis via regulating Pim-1 signaling. Mediators Inflamm.

[37] Fu R (2019). Pim-1 as a therapeutic target in Lupus Nephritis. Arthritis Rheumatol.

[38] Green JP (2018). Chloride regulates dynamic NLRP3-dependent ASC oligomerization and inflammasome priming. Proc Natl Acad Sci U S A.

[39] Sun Z (2022). Targeting macrophagic SHP2 for ameliorating osteoarthritis via TLR signaling. Acta Pharm Sin B.

[40] Glasson SS (2010). The OARSI histopathology initiative - recommendations for histological assessments of osteoarthritis in the mouse. Osteoarthritis Cartilage.

[41] Didichenko SA (2008). IL-3 induces a Pim1-dependent antiapoptotic pathway in primary human basophils. Blood.

[42] Miura O (1994). Induction of tyrosine phosphorylation of Vav and expression of Pim-1 correlates with Jak2-mediated growth signaling from the erythropoietin receptor. Blood.

[43] Huang B (2021). Ticagrelor inhibits the NLRP3 inflammasome to protect against inflammatory disease independent of the P2Y(12) signaling pathway. Cell Mol Immunol.

[44] Trachalaki A (2021). Enhanced IL-1β release following NLRP3 and AIM2 inflammasome stimulation is linked to mtROS in Airway Macrophages in Pulmonary Fibrosis. Front Immunol.

[45] Ahn H, Lee GS (2020). Riboflavin, vitamin B2, attenuates NLRP3, NLRC4, AIM2, and non-canonical inflammasomes by the inhibition of caspase-1 activity. Sci Rep.

[46] Zhou F (2019). Kinsenoside attenuates osteoarthritis by repolarizing macrophages through inactivating NF-κB/MAPK signaling and protecting chondrocytes. Acta Pharm Sin B.

[47] Sanchez-Lopez E (2022). Synovial inflammation in osteoarthritis progression. Nat Rev Rheumatol.

[48] Zhang B (2020). SQSTM1-dependent autophagic degradation of PKM2 inhibits the production of mature IL1B/IL-1β and contributes to LIPUS-mediated anti-inflammatory effect. Autophagy.

[49] Siddiqi S, Sussman MA (2013). Cell and gene therapy for severe heart failure patients: the time and place for Pim-1 kinase. Expert Rev Cardiovasc Ther.

[50] Wu T, Li Z, Wei Y (2023). Advances in understanding mechanisms underlying mitochondrial structure and function damage by ozone. Sci Total Environ.

[51] Zhou H (2022). Extracellular vesicles derived from human umbilical cord mesenchymal stem cells alleviate osteoarthritis of the knee in mice model by interacting with METTL3 to reduce m6A of NLRP3 in macrophage. Stem Cell Res Ther.

[52] Wu X (2019). The role of ca(2+) in acid-sensing ion channel 1a-mediated chondrocyte pyroptosis in rat adjuvant arthritis. Lab Invest.

[53] Wang T, He C (2018). Pro-inflammatory cytokines: the link between obesity and osteoarthritis. Cytokine Growth Factor Rev.

[54] Caso F (2022). Analysis of rheumatoid- vs psoriatic arthritis synovial fluid reveals differential macrophage (CCR2) and T helper subsets (STAT3/4 and FOXP3) activation. Autoimmun Rev.

[55] Ni L (2022). Itaconate attenuates osteoarthritis by inhibiting STING/NF-κB axis in chondrocytes and promoting M2 polarization in macrophages. Biochem Pharmacol.

[56] Elsaid KA (2015). The impact of early intra-articular administration of interleukin-1 receptor antagonist on lubricin metabolism and cartilage degeneration in an anterior cruciate ligament transection model. Osteoarthritis Cartilage.

