# Supplementary material for: Targeting macrophagic PIM-1 alleviates osteoarthritis by inhibiting NLRP3 inflammasome activation via suppressing mitochondrial ROS/Cl− efflux signaling pathway
Source: J Transl Med. 2023 Jul 8;21:452. doi: 10.1186/s12967-023-04313-1 (PMC10329339; doi:10.1186/s12967-023-04313-1)
Supplement: Supplementary file 1 — Additional file 1. Fig. S1. PIM-1 blockade inhibites the NLRP3 inflammasome activation in macrophages. Fig. S2. PIM-1 inhibitor AZD1208 inhibites the NLRP3 inflammasome activation in macrophages. Fig. S3. SMI-4a suppresses the NLRP3 inflammasome activation in macrophages. Fig. S4. SMI-4a inhibits the maturity of Cleaved-GSDMD in macrophages. Fig. S5. SMI-4a has no effect on the interaction of NLRP3 and ASC in macrophages. [file 12967_2023_4313_MOESM1_ESM.docx]

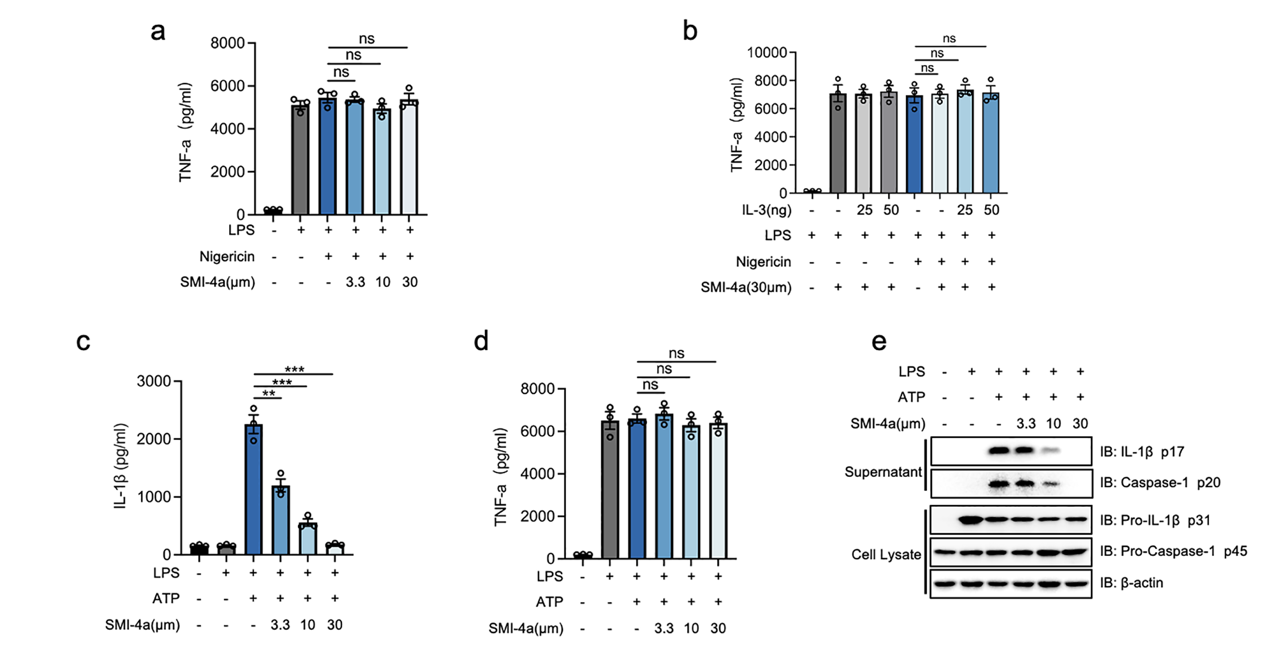


**Additional file 1: Fig. S****1: PIM-1 blockade inhibited the NLRP3 inflammasome activation in macrophages.**

(**a**) BMDMs were stimulated with LPS (1μg/mL) for 4h, whereafter co-incubated with SMI-4a in different doses for 2h, followed by 45 min of nigericin stimulation. The secretion levels of TNF-a. (**b**) LPS-primed BMDMs were treated with different doses of IL-3 for 2h, whereafter co-incubated with SMI-4a (10 μM) for 2h, followed by 45 min of nigericin stimulation. The secretion levels of TNF-a. (**c-e**) BMDMs were stimulated with LPS (1μg/mL) for 4h, whereafter co-incubated with SMI-4a in different doses for 2h, followed by 45 min of ATP stimulation. The secretion levels of IL-1β (**c**) and TNF-α (**d**). The protein levels of supernatant and cell extracts (**e**). Statistics in a, b,d, and e were performed using Student’s test. *P < 0.05, **P < 0.01, ***P < 0.001, ns P > 0.05. Bars represent mean ± SD. N = 3 experiments.

**
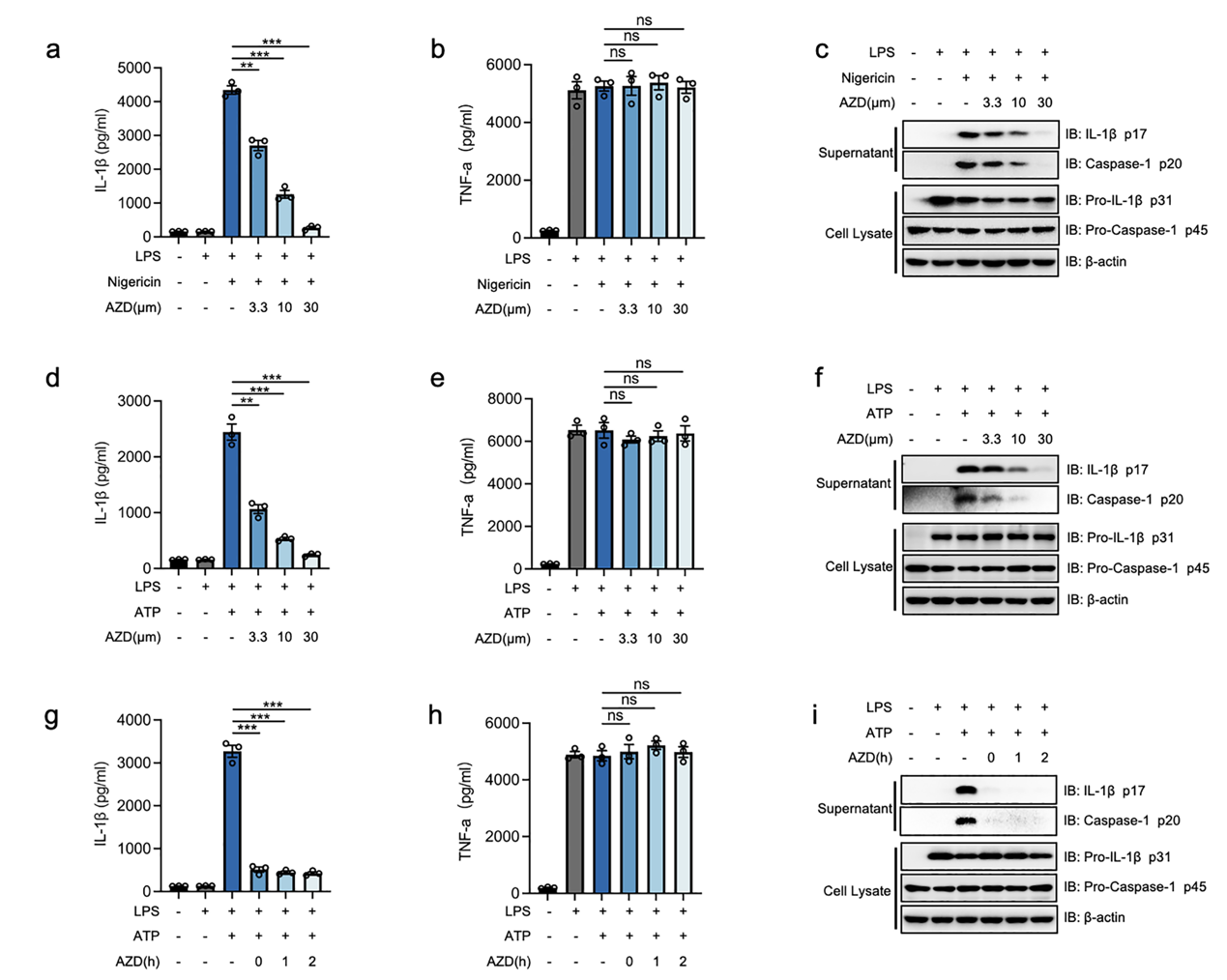
**

**Additional file 1: Fig. S2: PIM-1 inhibitor AZD1208 inhibited the NLRP3 inflammasome activation in macrophages**

**(a-f)** BMDMs were stimulated with LPS (1μg/mL) for 4h, whereafter co-incubated with AZD1208 in different doses for 2h, followed by 45 min of nigericin **(a-c)** or ATP **(d-f)** stimulation. The secretion levels of IL-1β **(a and d)** and TNF-α **(b and e).** The protein levels of supernatant and cell extracts **(c and f)**. **(g-i)** BMDMs were stimulated with LPS (1μg/mL), whereafter co-incubated with AZD1208 (30 μM) at different times, followed by 45 min of nigericin stimulation. The secretion levels of IL-1β **(g)** and TNF-α **(h).** The protein levels of supernatant and cell extracts **(i)**. Statistics in a, b, d, e, g, and h were performed using Student’s test. *P < 0.05, **P < 0.01, ***P < 0.001, ns P > 0.05. Bars represent mean ± SD. N = 3 experiments.

**
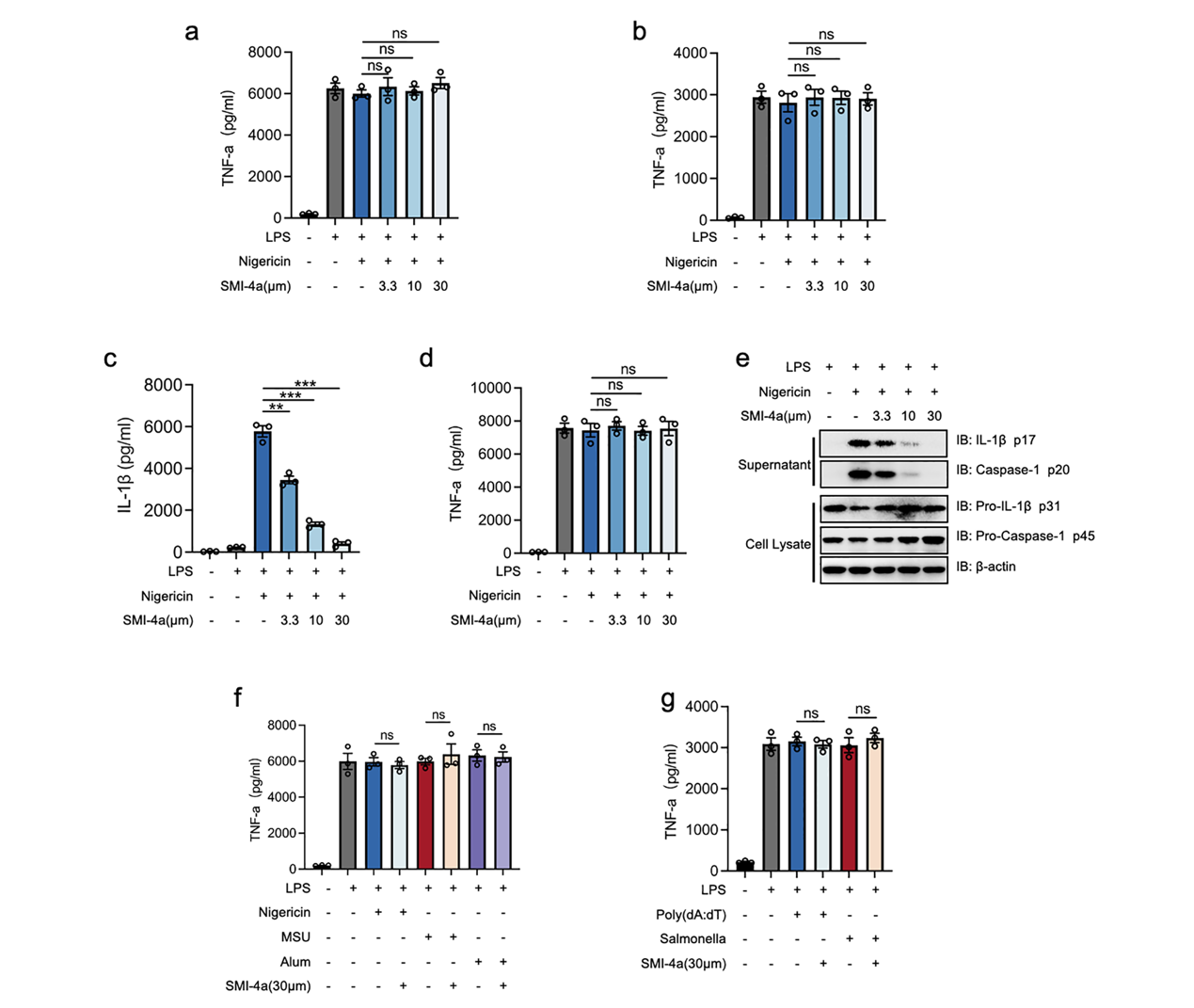
 Additional file 1: Fig. S3: SMI-4a suppressed the NLRP3 inflammasome activation in macrophages.**

(**a-b**) PMs from C57BL/6 mice (**a**) and THP-1 (**b**) were stimulated with LPS (1μg/mL) for 4h, whereafter co-incubated with SMI-4a, followed by 45 min of nigericin stimulation. The secretion levels of TNF-α. (**c-e**) LPS-primed PBMC from healthy people were treated with different doses of SMI-4a and then stimulated with nigericin for 45 min. The secretion levels of IL-1β (**c**) and TNF-α (**d**). The protein levels of supernatant and cell extracts (**e**). (**f**) BMDMs were stimulated with LPS (1μg/mL) for 4h, whereafter co-incubated with SMI-4a (30 μM), and then challenged by nigericin for 45 min, MSU and Alum for 3h. The secretion levels of TNF-α. (**g**) BMDMs were stimulated with LPS (1μg/mL) for 4h, whereafter co-incubated with SMI-4a (30 μM) for 2h, followed by handled with poly (dA:dT) transfection or Salmonella infection. The secretion levels of TNF-α. Statistics in a-d, f, and g were performed using Student’s test. *P < 0.05, **P < 0.01, ***P < 0.001, ns P > 0.05. Bars represent mean ± SD. N = 3 experiments.


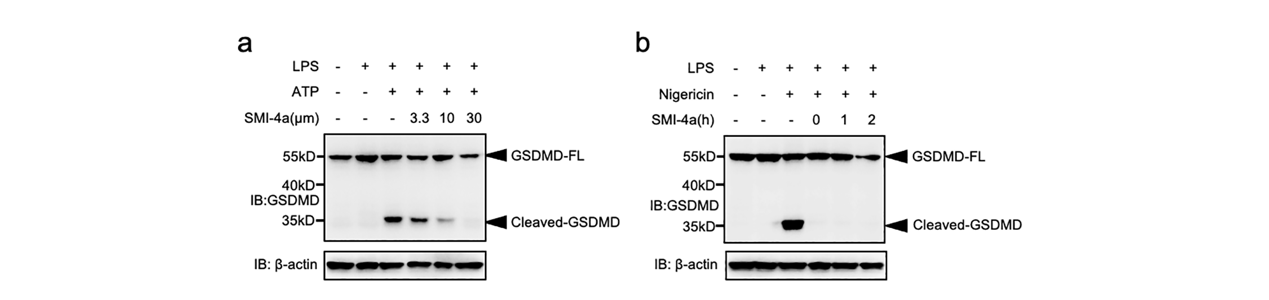


**Additional file 1: Fig. S4: SMI-4a inhibits** **the maturity of Cleaved-GSDMD in macrophages.**

(**a**) BMDMs were stimulated with LPS (1μg/mL) for 4h, whereafter co-incubated with SMI-4a in different doses, followed by 45 min of ATP stimulation. Cell extracts were analyzed by western blot. (**b**) BMDMs were stimulated with LPS (1μg/mL) for 4h, whereafter co-incubated with SMI-4a at different times, followed by 45 min of nigericin stimulation. Cell extracts were analyzed by western blot. N = 3 experiments.


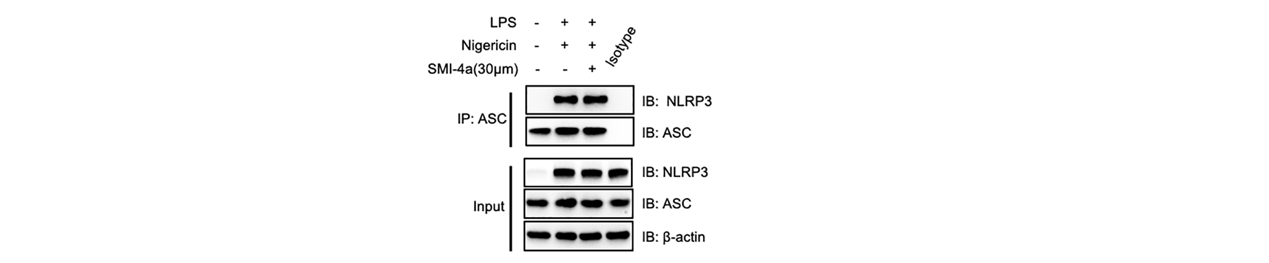


**Additional file 1: Fig. S5: SMI-4a has no effect on the interaction of NLRP3 and ASC in macrophages.**

BMDMs were stimulated with LPS (1μg/mL) for 4h, whereafter co-incubated with SMI-4a (30 μM), followed by 45 min of nigericin stimulation. The NLRP3-ASC interaction was analyzed by co-immunoprecipitation and western blot. N = 3 experiments.
